# Anticonformists catalyze societal transitions and facilitate the expression of evolving preferences

**DOI:** 10.1093/pnasnexus/pgae302

**Published:** 2024-07-25

**Authors:** Dhruv Mittal, Sara M Constantino, Vítor V Vasconcelos

**Affiliations:** Computational Science Lab, Informatics Institute, University of Amsterdam, 1098 XH, Amsterdam, The Netherlands; School of Public and International Affairs, Princeton University, Princeton, NJ 08544, USA; Department of Psychology, Northeastern University, Boston, MA 02115, USA; School of Public and Urban Affairs, Northeastern University, Boston, MA 02115, USA; Doerr School of Sustainability, Standford University, Stanford, CA 94305, USA; Computational Science Lab, Informatics Institute, University of Amsterdam, 1098 XH, Amsterdam, The Netherlands; POLDER, Institute for Advanced Study, University of Amsterdam, 1012 GC, Amsterdam, The Netherlands

**Keywords:** complex adaptive systems, collective behavior, network effects, social norms, conformity traps

## Abstract

The world is grappling with emerging, urgent, large-scale problems, such as climate change, pollution, biodiversity loss, and pandemics, which demand immediate and coordinated action. Social processes like conformity and social norms can either help maintain behaviors (e.g. cooperation in groups) or drive rapid societal change (e.g. rapid rooftop solar uptake), even without comprehensive policy measures. While the role of individual heterogeneity in such processes is well studied, there is limited work on the expression of individuals’ preferences and the role of anticonformists—individuals who value acting differently from others—especially in dynamic environments. We introduce anticonformists into a game-theoretical collective decision-making framework that includes a complex network of agents with heterogeneous preferences about two alternative options. We study how anticonformists’ presence changes the population’s ability to express evolving personal preferences. We find that anticonformists facilitate the expression of preferences, even when they diverge from prevailing norms, breaking the “spiral of silence” whereby individuals do not act on their preferences when they believe others disapprove. Centrally placed anticonformists reduce by five-fold the number of anticonformists needed for a population to express its preferences. In dynamic environments where a previously unpopular choice becomes preferred, anticonformists catalyze social tipping and reduce the “cultural lag,” even beyond the role of committed minorities—that is, individuals with a commitment to a specific cause. This research highlights the role of dissenting voices in shaping collective behavior, including their potential to catalyze the adoption of new technologies as they become favorable and to enrich democracy by facilitating the expression of views.

**Significance Statement**

Perceptions of social norms are important in shaping individual attitudes and behaviors. They can reinforce the status quo even when underlying preferences diverge or changing contexts render alternative options preferable. People’s tendency to conform to the norms they perceive can create a reinforcing cycle where individuals hesitate to share or act on their own preferences when they believe others disapprove. This spiral of silence maintains social norms despite diverging preferences or contribute to widespread misperceptions of social norms, as shown across many domains. Here, we show that anticonformists—individuals who earn utility from acting differently from others—help groups overcome new conformity traps by encouraging the expression of counter-normative views, though this depends on where they exist in social networks.

## Introduction

Coordinated collective action is necessary to mitigate the worst impacts of climate change (1). Scientific reports, such as the IPCC, identify household behavioral change as a sizeable lever for reducing a country’s carbon emissions (2). Individual decisions are influenced by myriad considerations, including personal material outcomes, knowledge, and social factors. In recent years, there has been a growing interest in leveraging social influence to bring about behavioral changes on large scales (1, 3). Such an approach has the potential to minimize the extent of top-down interventions while driving rapid social change, as reinforcing feedback can lead to the existence of social tipping points (4–6). However, while social influence can be a force for rapid change, it can also entrench the status quo. This can create two challenges: (1) social systems may be slow to respond to changing environments due to the inertia created by social influence and (2) collective misperceptions of social norms can sustain behavioral equilibria even when they diverge from underlying preferences (7).

One reinforcing feedback considered in studies on social tipping is the direct or indirect incentive that individuals face to act like others, equivalent to those of a coordination game (4, 5, 8). In many circumstances, people show a strong tendency to conform to the behaviors they perceive around them, even when those behaviors or beliefs contradict their personal preferences (9–11). This type of conformity has been described as a bias where people show a disproportionate tendency to follow the majority (12). Aligning with others can also be a heuristic or social learning strategy to account for social information in decision-making (13). Conformity can arise out of trust and cohesiveness or even out of fear of ostracism or ridicule (14).

While the pressure to conform can precipitate consensus on matters where people hold differing opinions and can help to sustain cooperation and prosocial behaviors in the face of incentives to act in one’s own self-interest, it may also lead to phenomena such as “groupthink” (15, 16) and the suppression of diverse or heterodox ideas by creating a “spiral of silence” (17). The hesitancy to express one’s views could erode the democratic process, which depends on the expression of individual opinions (16, 18). In the context of social learning, rewarding originality can reduce herding behavior (19). However, groupthink can occur when a group’s members are reluctant to publicly express private concerns about collective behaviors if they believe that other members are likely to disagree with them. This can, in turn, lead to pluralistic ignorance—the shared and systematic misperception of the true beliefs or actions in a population (14, 20, 21). For example, recent studies suggest that widespread misperceptions about the beliefs or actions of others, such that those with proclimate positions perceive themselves to be in the minority despite being in the majority, can stifle climate action by both the public and policymakers (22, 23). Misperceptions may also sustain collectively desirable behaviors. For example, a recent study found that the expression of xenophobic views became quickly acceptable in some communities following the 2016 US election, suggesting that people became more inclined to express views that they had previously perceived to be stigmatized when a public signal made visible prevailing preferences in a population (24). Here, we remain agnostic as to whether the expressed norm is desirable or not and focus instead on the role of anticonformists in facilitating the expression of underlying preferences in the face of pressures to conform.

Perhaps most importantly, social conformity can lead to a cultural lag (25). A cultural lag can occur when the broader environment changes quickly relative to changes in the social system. The tendency to conform to or coordinate with others can create social inertia that slows the ability of a society to adapt or respond to changes (7). This is particularly relevant in the rapidly evolving context of climate change, which has introduced new climactic patterns, including increased flooding and fires, and will require rapid and expansive changes to our physical systems, but also economic and cultural aspects of life. Social inertia may delay the adoption of sustainable technologies, like electric vehicles or heat pumps, and lifestyle choices, like vegetarian diets. In such contexts, dissenting voices that perturb the status quo may actually help keep society in step with such changes (26, 27). In this paper, we focus on a specific type of dissenting voice: anticonformists. Anticonformists are individuals who, all else equal, experience a utility from acting antinormatively.

Evidence of anticonformity in human behavior has been observed in various contexts (28–31). This tendency has also been observed in nonhuman species (32). The Optimal Distinctiveness Theory offers a valuable framework for understanding the motivations behind anticonformity at the individual level (33). This theory posits that individuals have two contrasting psychological needs: the need for inclusion and the need for differentiation or uniqueness. The need for inclusion drives individuals to conform and seek acceptance within their social groups, while the need for differentiation pushes them to assert their uniqueness and individuality. Anticonformists represent individuals who prioritize the need for differentiation. Anticonformity corresponds to incentives present in the anticoordination or chicken games, in which individuals benefit from acting differently from others (34, 35). Supply and demand effects can also generate utility from anticoordination (36). What makes anticonformity particularly intriguing is its potential for disproportionate influence on population dynamics compared to the relative number of anticonformists in the population (37, 38).

**Fig. 1.**
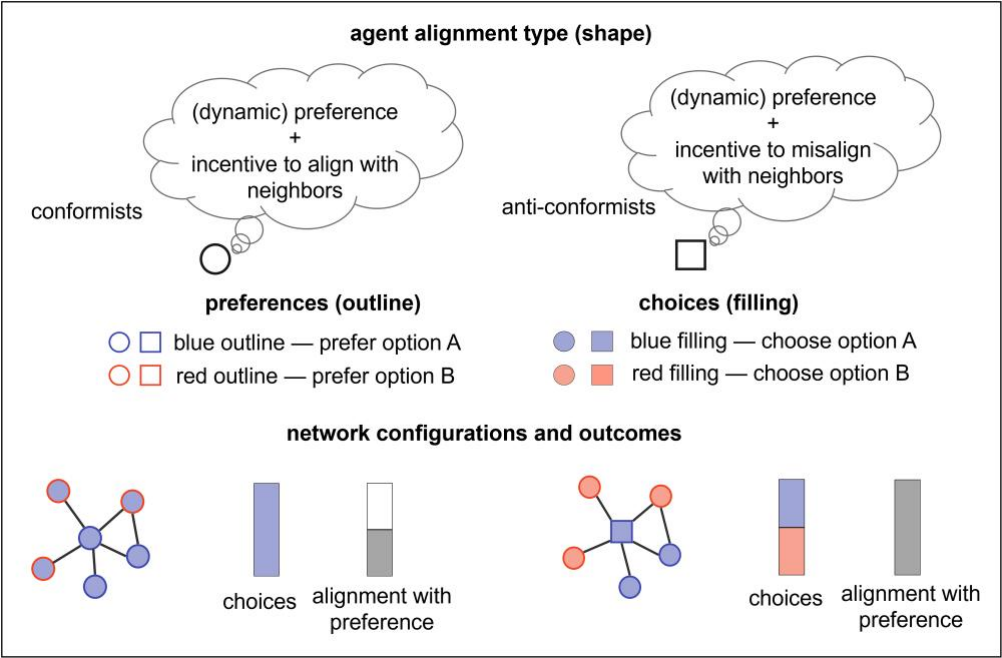
Stylized model. Agents have preferences for option A (blue outline) or B (red outline), and these preferences can change over time. There are two types of agents: conformists or anticonformists. Conformists (circles) earn utility from acting like others in their networks, while anticonformists (squares) earn utility from acting differently. These social dynamics mean that choices (filling) can diverge from underlying preferences (outlines), and can result in a conformity trap. In this diagram, we show how the presence of an anticonformist can facilitate the expression of underlying preferences, and increase alignment between choices and preferences.

To understand the collective dynamics of preference expression and social change in dynamic environments, one must consider the complexities of social systems. However, there are limited parsimonious theoretical frameworks to describe social dynamics in heterogeneous settings (39). Moreover, while most studies on collective decision-making focus on consensus dynamics, few study the expression of individual preferences under social influence (40). To address this gap, we take a bottom-up approach and present a micro-mechanistic framework of collective decision-making based on game theory that considers diverse preferences, social conformity, and network effects—three important features of social systems with implications for how social change or inertia starts and whether it spreads (Fig. 1). Building on previous work, we define conformity as a frequency-dependency of the focal agent’s decision on surrounding states (10, 28). We use this framework to explore the following two questions.

What impact do anticonformists have on the overall expression of underlying preferences in a society where most actors receive most utility from conforming to prevailing social norms? Under which conditions does the presence of anticonformists facilitate or hinder the responsiveness of collective decision-making to dynamic or changing environments?

Our research demonstrates that anticonformists play a significant role in facilitating the expression of preferences in a population, especially when they are centrally situated within a network. Additionally, we show that in a changing environment, anticonformists can increase the adaptability of a population, though only up to a point. Thus, anticonformists can overcome conformity traps, though there exists an optimal fraction of anticonformists beyond which the benefits of anticonformity diminish.

## Results

Consider a population that must choose between two options, A and B. For example, these could be competing technologies like electric or fossil-fuel cars or deciding whether or not to wear a face mask during a pandemic. Individuals differ in their underlying preferences, and these preferences can evolve over time as the costs and quality of the options change. Additionally, individuals can gain or lose marginal utility (ΔU) by conforming to those around them, though there is heterogeneity in how much they value (or dislike) conformity. Thus, choices are determined by a combination of personal preferences ($o_{A},o_{B}$ for option A and B, respectively) and utility gained from both alignment or misalignment with locally perceived social norms (refer to Eq. 1 in the Materials and methods section and Supplementary material for a more general framework). Agents obtain information about social norms from the choices of neighbors who are visible or connected to them in a social network. Agents can only observe the choices of their neighbors to gauge the descriptive social norm without having information about the preferences, conformity, or their neighbors’ immediate network. A conformist’s (anticonformist’s) probability of picking a choice, given by Eq. 2, increases (decreases) as more neighbors adopt that choice (Fig S1). We also consider a different type of agent, which we call nonconformists, whose decision-making is solely based on their preferences—that is, they do not consider local social norms in making their decisions. We start with fixed individual preferences but allow these to evolve in dynamic environments. We consider populations that are connected in random (Erdős–Rényi, ER) and highly heterogeneous (Barabási–Albert, BA) social networks. For large populations, the number of connections of ER networks follows a Poisson distribution, whereas for the BA networks, they are scale-free. These networks allow us to mimic real-world social network properties like small geodesic distances (small-world property), high clustering, and the presence of hubs. We also discuss and quantify different measures of social well-being, including social welfare, in Section 5 of the supplementary material. We show that while the utility of alignment increases with the fraction of anticonformists, total welfare can increase or decrease for two populations with identical decision-making.

### Expression of preferences

First, we check for the equilibrium alignment between the choices and preferences of individuals for populations on BA networks, where half the population prefers A ($\Delta o\equiv o_{A}-o_{B}=10$) and the other half prefers B (Δo=−10). For simplicity (and because previous work has shown that modality is the main driver of the dynamics (41, 42)), we assume homogeneity of preference within each sub-population. Since the utility is also a function of node degree, the heterogeneity of node degree ensures heterogeneity in the distribution of the effective threshold fraction of neighbors within each sub-population required to make individuals pick a different option. We first consider that the strength of preferences for either choice remains constant. We randomly place anticonformists in the network and randomly assign choices and personal preferences to individuals corresponding to different assumptions about the initial fraction of As in a population.

**Fig. 2.**
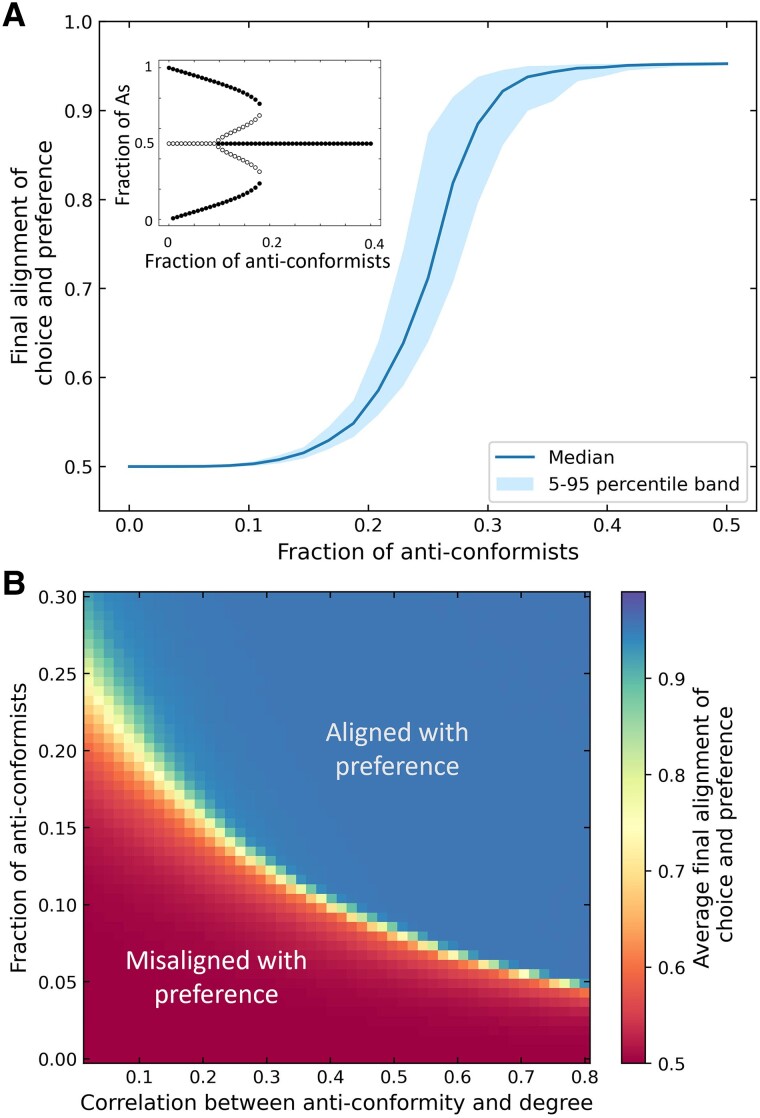
Expression of preferences: The average equilibrium alignment of choices and preferences are plotted for a heterogeneous population comprising half of individuals who prefer A and half who prefer B. Agents were placed on a BA network with $k_{\min}=20$. A) For the heterogeneous BA network, there is a strong nonlinear continuous response to the fraction of anticonformists in the population driven by local network effects. In networks without spatial correlations with probabilistic connections (inset and Fig S8 and a detailed description of these networks in the supplementary materials) and ER networks (Fig. S10), the transition is discontinuous in the fraction of anticonformists. B) A sharp phase transition is seen from a state of misalignment between the choices and preferences to a state of alignment in the parameter space as the centrality and fraction of anticonformists increase. Populations with a low fraction of anticonformists achieve alignment between choice and preference only when anticonformists are more central in the network. The simulations are done for population size 2,000 with 100 different network realizations.

For high enough conformity pressure, we find that populations with few anticonformists persist on the initially dominant choice (Fig. 2A), despite even underlying preferences for the two options. This leads to widespread misalignment or divergence between preferences and choices. As the fraction of anticonformists in the population increases, the population is more likely to express their preferences, and this increases sharply around the critical number of anticonformists computed analytically in the well-mixed setting (see the inset in Fig. 2A) but is affected by locality effects. The dynamics of the fraction of the population adopting A around the critical point slows down considerably, which is indicative of the *critical slowing down* present in the well-mixed system (43). This is accompanied by a sharp increase in volatility in the population around the critical value (Fig. S14). Volatility is defined as the number of individuals changing their choices after an initial period—a potential early warning signal for social tipping points (38). We find similar results for ER networks (Fig. S15).

Next, we examine how the choice dynamics change as anticonformists are strategically placed in a network. Specifically, we check the effect of degree centrality of the anticonformists by varying the correlation between node degree and anticonformity. When anticonformists are more central in the network, we observe a five-fold decrease in the number of such agents required for the population to express their preferences (Fig. 2B). The critical value of correlation between degree and conformity for a given fraction of anticonformists is fairly robust to populations with differently skewed preference distributions (Fig S16). These results highlight the important role of high-degree nodes or hubs in shaping social dynamics.

### Adapting to dynamic environments

Next, we examine how the choices in a population change as the underlying preferences evolve. Changes in preferences arise due to the changing utility of choices, which could be due to falling prices (e.g. due to subsidies or tax credits) or increases in quality. In this computational experiment, all individuals share the same preference, which evolves as a function of time. Thus, we exclude heterogeneous preferences from the present analysis. We examine two cases of evolving preferences: a linear decrease in the utility of A relative to B and a sinusoidal fluctuating utility that cycles back and forth between A and B. The linear scenario captures cost reductions of new technologies, like rooftop solar panels. In contrast, sinusoidal utility can represent preferences for options that fluctuate in their utility such as inconsistent policies or wearing a face mask as disease prevalence ebbs and flows during a pandemic.

**Fig. 3.**
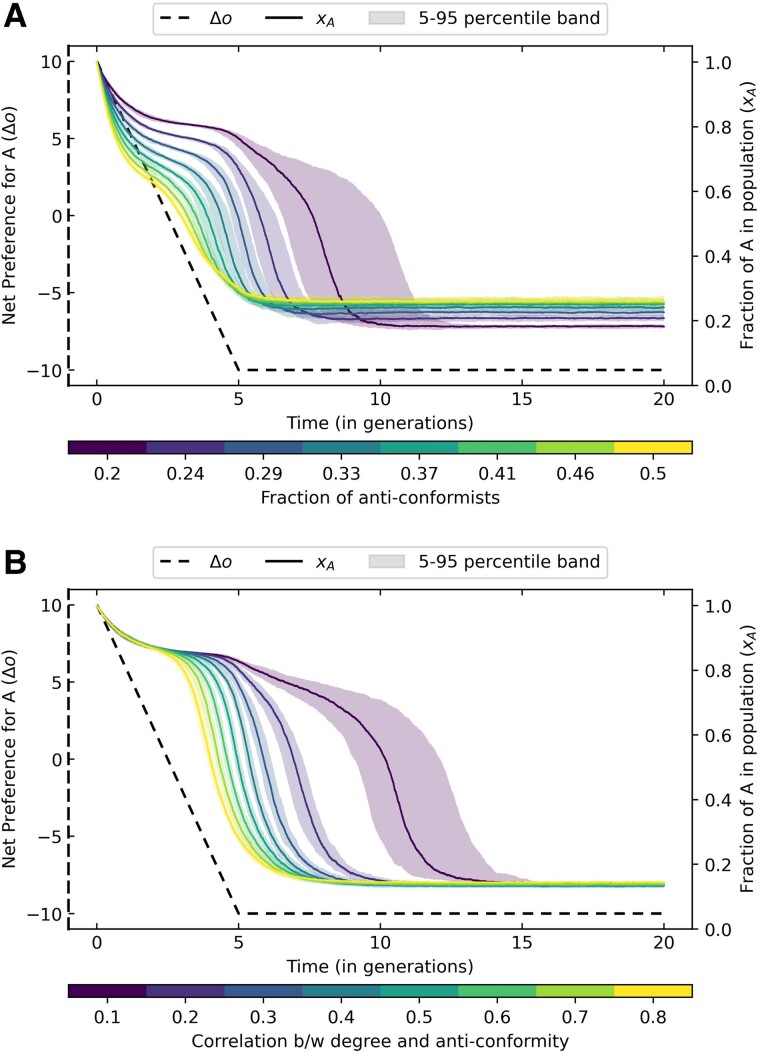
Dynamic environments: The net preference for choice A (Δo) decreases linearly with time. In both figures, we plot the median trajectory of the fraction of choice A in the population on BA networks over time. In the top panel, we vary the fraction of anticonformists in the population. In the bottom panel, we vary the degree centrality of anticonformists in the population, keeping the fraction of anticonformists equal to 0.15. We see a faster societal transition from choice A to B as the fraction and degree-centrality of anticonformists increases. The population size is fixed at 1,000.

When preferences change linearly, we find that a population can remain stuck or trapped in a choice equilibrium they no longer prefer when conformity is high enough. The introduction of anticonformists can drive social tipping, leading the population to rapidly transition to the newly preferred choice (Fig. 3A). The time taken for social tipping decreases rapidly as the fraction of anticonformists in a population increases (Fig S11 A) This qualitatively matches theoretical expectations (Fig S17). The anticonformists emerge as instigators of change, and they are followed by the conformists. Thus, anticonformists can mitigate conformity traps by reducing the social pressure for the initial option (Fig S11 B). However, as the fraction of anticonformists increases, the fraction of the population choosing the suboptimal or nonpreferred choice also increases. This leads to the existence of an optimal fraction of anticonformists in a population beyond which the population’s satisfaction decreases (Fig S11 C). Above the optimal fraction, the average alignment of choice and preference over time for the population plateaus (Fig S11 D).

In Figure 3B, we test the effect of the centrality of anticonformists in a population connected by a BA network. We vary the correlation between degree and anticonformity for a fixed fraction of anticonformists in the population. We find that a greater degree-centrality of anticonformists in the population leads to faster social tipping. We also explore the effect of degree assortativity of networks, i.e. the tendency of high-degree nodes to be connected to other high-degree nodes, on social tipping in setups with centrally placed anticonformists. Assortative networks take more time to tip compared to neutral and disassortative networks when anticonformists are centrally placed (Fig S18).

**Fig. 4.**
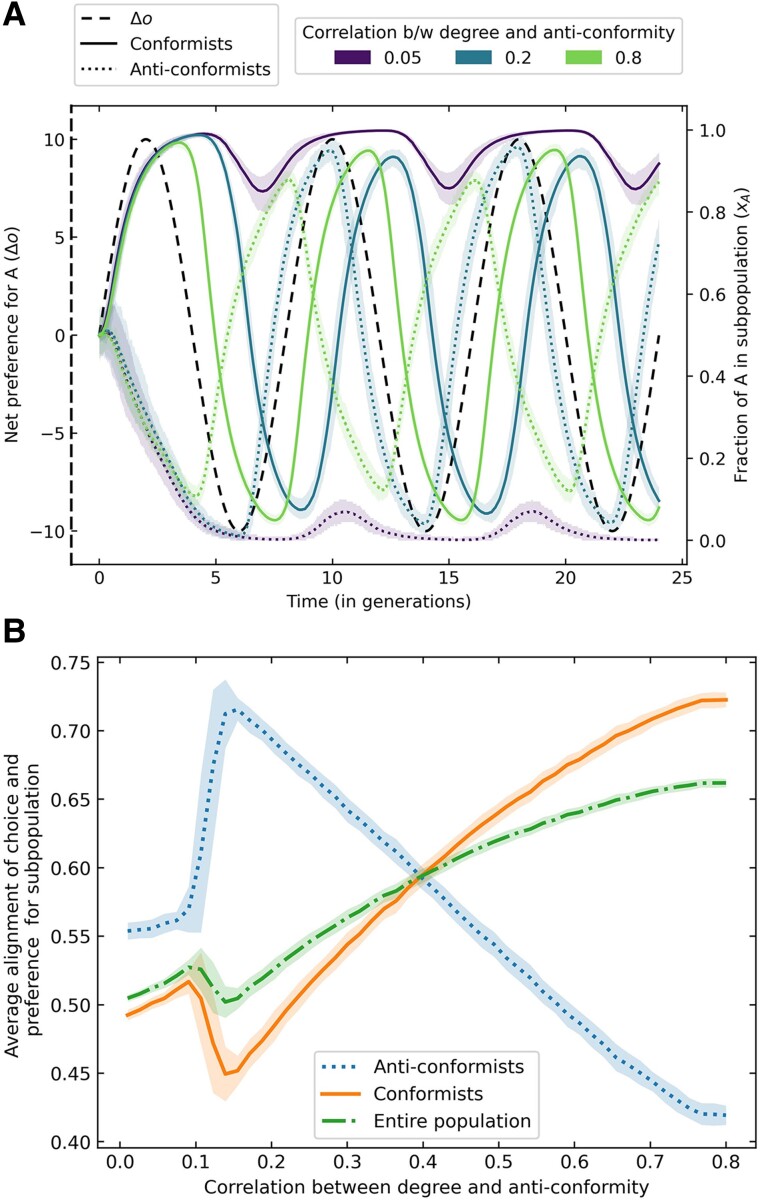
Fluctuating environments: The net preference for choice A (Δo) fluctuates sinusoidally with time. In the top panel, we plot the median trajectory of the fraction of different sub-populations, i.e. the conformists (solid lines) and anticonformists (dotted lines), picking choice A over time. Colors indicate populations that vary in the value of the correlation between degree and anticonformity, keeping the fraction of anticonformists equal to 0.2. As anticonformists become more central, they switch their choices to the alternative behavior or technology before it is even profitable. This allows the conformists’ choices to track better the changes in preferences $o_{A}$ (dashed line). The bottom panel shows the average alignment between the choices and preferences of the two sub-populations and for the entire population over time as a function of differing degree centrality of anticonformists in the population. The population size considered is fixed at N=1,000.

In Figure 4, we consider agents with fluctuating preferences due to a changing environment (e.g. changing policies, disease waves in a pandemic, or a natural resource with productive and barren periods). Here, we assume that preferences update quickly as the environment changes, but may not be expressed due to social dynamics reinforcing the status quo. Here, we find again that the population choices track the underlying preferences better when there are more centrally placed anticonformists in the population. Once more, anticonformists emerge as leaders of change, staying ahead of the curve by switching their choices before the alternative choice becomes optimal (Fig. 4A). This behavior, which is driven by their preference for distinctiveness, reduces the time lag for conformists by increasing the heterogeneity of the social influence in their networks, allowing them to express choices that are aligned with their preferences. As anticonformists become more central in a network, they tend to switch their choices earlier, resulting in lower average payoffs for anticonformists (Fig. 4B). However, this leads to increased payoffs for the rest of the population, i.e. the conformists, as their choices are more in sync with the environment. In comparison, nonconformists fail to create such an effect because their decisions coincide with the changing preferences, which leads to the conformists’ choices lagging behind environmental changes (Fig. S7).

## Discussion

In this study, we consider situations where the decisions of others are visible and have public externalities and are, therefore, rife for social conformity and coordination dynamics. For example, this might include mask-wearing during a pandemic, use of politically correct language, adoption of electric vehicles or rooftop solar, or individual catch in a fishery. In such situations, individuals may conform to perceived social norms, despite personal preferences that diverge from the perceived norm, in order to avoid disapproval from others (41, 42).

Our findings underscore the nuanced influence of anticonformists within social networks. By valuing distinctiveness, anticonformists can create the social license for more conforming types to express nonnormative opinions or actions. Thus, their presence can break the “spiral of silence” and enable a more authentic representation of societal preferences. Further, they can facilitate the expression of preferences in dynamic environments, where preferences evolve with changing circumstances but norms are slow to change due to social inertia.

Our study assumes agents have heterogeneous personal preferences that evolve as background conditions change. For example, new technologies such as electric vehicles and rooftop solar often have steeply decreasing costs (e.g. due to technological learning, returns to scale, government subsidies) and increasing quality. Other decision contexts, such as mask-wearing, natural resource use, or public transit, may instead show cyclical patterns depending on disease rates and resource abundance (42, 44). Their preference may include heterogeneous injunctive, nonlocal norms. Our agents consider their personal preferences and the local descriptive norms in their networks when making a choice—together, these generate a distribution of thresholds in the population that determine how individuals in a population change their behaviors in response to changes among those in their networks. Threshold models have been traditionally used to describe collective behavior (4, 45). Additionally, our approach to modeling social dynamics incorporates agents with a range of conformist and anticonformist tendencies within realistic network structures, allowing for a nuanced exploration of how individual preferences, choices, and position in a network impact collective decision-making. In the Supplementary material, we show that the effect of anticonformists is maintained (to a lesser extent) even when their behavior is more nuanced. There, we consider individuals who, like all those considered in the main text, still have preferences and, like the anticonformist, change to a minority behavior when surrounded by a large majority. In the absence of a large majority, they are more similar to conformists, i.e. they are inclined to conform with others and require less reinforcement from their preference to express it.

Anticonformists in our model represent individuals who experience a disutility from adopting the choice that most others in their network have adopted (Fig S1). They are similar to hipsters in the study by Juul and Porter (46), and differ from the “bias” interpretation of anticonformity, also called “weak conformity” (47), where the adoption probability remains monotonically increasing with the salience of each option (10). Such a definition would lead to different nonlinear individual conformist responses but identical social dynamics, with additional potential social traps (20). Anticonformists also differ from committed minorities—individuals who are committed to a specific nonnormative behavior (e.g. activists). Committed minorities have been shown to tip societies from a status quo to an alternative state in theoretical and experimental settings (4, 45, 48). In contrast, anticonformists are not committed to a specific outcome but instead prefer to go against the tide, whatever the tide is. Thus, they may be essential in dynamic environments, especially cyclical ones.

Compared to existing research, our study explicitly models the interplay between conformist and anticonformist behaviors within heterogeneous networks with explicit preferences. Studies considering anticonformity have been restricted to well-mixed systems (49–52). Our analysis benefits from a robust methodological framework that combines analytical techniques with agent-based modeling, offering insights into the critical role of networks, individual centrality, and the optimal fraction of anticonformists for societal adaptability in dynamic environments. While studies have looked at collective outcomes in the context of social learning, the expression of preferences in contexts with strong social norms or pressure to conform remains under-explored (40). Further, only a few studies have examined collective decision-making in dynamic environments where the utility associated with different choices might change gradually or rapidly, and linearly or cyclically (53).

Several studies have shown that the tendency of individuals to conform to others can create conformity traps. Conformity traps refer to situations where social reinforcement is strong enough to sustain suboptimal choices (54). Anticonformists can not only help a population abandon a suboptimal choice but also return to that choice if it becomes optimal again. The population response becomes better synchronized with the environment. Anticonformists can affect this change by staying ahead of the curve as they always oppose the status quo. While the opposition to the status quo can come at an (immediate) personal cost to anticonformists, it can benefit collective outcomes. Thus, whenever the optimality of the status quo falls, they emerge as leaders or trendsetters. This ability distinguishes anticonformists from stubborn agents or committed minorities who do not respond to social pressure but rather make choices based on the direct utility associated with an outcome. Even though such nonconforming agents can switch their own choices in sync with the environment, it can be too late for the rest of the population to follow their lead. This can be very costly in a rapidly changing or fluctuating environment as this lag can lead a population to become out of sync with the environment. Further, a population requires about only half as many anticonformists as nonconformists to be able to express themselves much better (Fig. S6). This shows that it is not enough to be well-informed or committed to a cause to bring about desirable societal shifts. How individuals interact with their social environments is essential to the adaptability and resilience of a collective decision-making process.

Previous studies suggest that the critical mass required for social tipping is around 25% (48, 55), and can vary from 10 to 45% depending on the context, i.e. the heterogeneity of preferences and network structures (56). However, empirical studies also find that a much smaller critical fraction, i.e. 3.5%, can lead to the abandonment of the status quo (57). Our results show that when anticonformists are highly visible in a population, they can multiply the momentum of dissent and significantly reduce the number of anticonformists required to increase the adaptability of the collective decision-making process.

The assumption of static individual preferences within the model may only partially capture the complexity of real-world decision-making at shorter timescales, where preferences evolve slowly in response to new information or changing social cues. Additionally, focusing on anticonformity without considering the potential for subgroup formation or identity-driven behaviors likely oversimplifies the social dynamics (58). For example, in our model, individuals cannot identify the internal factors that underlie the choices of others (i.e. conformity, direct preferences), they only observe their choices. The ability to identify types could be modeled through a process of learning in a multiagent context (59). If agents are able to identify other agents’ attributes, prejudiced agents can choose to align with, disregard, or even diverge from the choices they associate with certain types of actors (60). Including the dimension of identity, segregation, and network coevolution would have a significant impact on collective decision-making (61–64). Thus, if conformists were to disregard the decisions of anticonformists, the latter would move to the periphery of the network or become effectively segregated, and, as the results suggest, it would subsequently render the conformists incapable of expressing their preferences and adapting to changing environments.

Our findings elucidate anticonformists’ role as catalysts of change and the expression of underlying preferences, even when they diverge from prevailing norms and as circumstances change. If a large part of the population has socially undesired preferences—preferences for options that harm the collective in the long term—the expression of preferences can lead to the emergence of undesirable social norms (65). As socially desirable preferences emerge, an adaptable society can more easily express them. For policymakers, this underscores the importance of not only leveraging social influence to promote rapid social change but also considering when it may be beneficial to reduce the social pressure to conform and maintaining communication about the collective desirable and undesirable outcomes of specific options. Clinicians and social scientists might leverage these insights to promote healthier social dynamics, encouraging individuality (44) or reflection about the feelings triggered by norm deviation (66) to enrich societal diversity and resilience. Further, our findings have implications for the adaptability and resilience of more conformist or “tight” societies, which may also face greater inertia when environments and preferences change (42, 67).

While our study suggests that anticonformity can have some advantages (at least at the group level), especially in changing environments, the evolution of conformity as a social learning strategy remains an area for future investigation. Additionally, exploring the feedback loop between collective decisions and environmental changes could offer deeper insights into how societies can more effectively navigate the complexities of sustainable development and technological adoption. Further research could also benefit from incorporating memory and identity-driven behaviors into models, enhancing the external validity and applicability of the findings to real-world scenarios (42, 58).

## Materials and methods

### Computer simulations

We develop an Agent-Based Model of collective behavior with binary options, A and B (68). Agents have preferences for A and B, $o^{A}$ and $o^{B}$, which are not visible. Δo, i.e. $o^{A}-o^{B},$ denotes the net preference the agent has for A. Choices are informed by a combination of direct preferences (e.g. material benefits or costs associated with a choice) and social influence. The choices made by neighbors are visible to the focal node. Thus, this model is applicable for choices that are visible rather than behaviors that are private. The conformity (*w*) of the agent determines how she incorporates social information, i.e. the number of neighbors choosing A(${\#}^{A}$) and B(${\#}^{B})$, in her decision-making. Each agent, *i*, calculates a marginal utility, $\Delta U_{i}$, when making a choice.


(1) $$\Delta U_{i}=\Delta o_{i}+w_{i}({{\#}_{i}}^{A}-{{\#}_{i}}^{B}).$$

In this game theoretical setting, the marginal utility is understood as an internal metric of decision-making. The agent uses the Fermi update rule (69), in which it chooses A with probability ${\;p_{i}}^{A}$ (and chooses B with probability $1-{\;p_{i}}^{A}$), with


(2) $${\;p_{i}}^{A}=1/(1+e^{-\Delta U_{i}\beta })$$

where we set the inverse temperature *β* to 100, effectively eliminating noise from the decisions and bringing the model closer to the threshold models in the literature. Individual choices are updated asynchronously, i.e, at each time step a random agent is picked from the population, evaluates its options, and decides whether to change states. This update rule mimics continuous time. Agents can start with either choice and are free to change the choice should the situation arise. The dependence of the utility on the difference $\left({\#}^{A}-{\#}^{B}\right)$ reflects the competing nature of the two choices. The final alignment of choices with direct preferences is calculated once equilibrium is reached. While we have assumed a linear frequency dependency, we also present a generalized model in the Supplementary material to consider higher-order dependencies to make agents’ behavior more nuanced (refer to the Supplementary material).

### Social networks

We have considered different synthetic networked populations: ER network and BA network. To generate networks with varying correlations between degree and anticonformity, we start with anticonformists occupying all the highest degree nodes, and then iteratively swap positions of two randomly selected individuals until we reach the desired correlation between degree and anticonformity. We conducte simulations for large population sizes to minimize finite-size effects. Assortative networks are generated using the algorithm used in (70).

### Analytical methods

We complement the ABM with analysis using Markov chains and calculate the transition probability of the macro-state using a mean-field approach based on the probabilities of switching choices in all the different sub-populations. Using the transition probability, we then identify the corresponding stable and unstable fixed points of the system with different configurations and parameters.

## Supplementary Material

pgae302_Supplementary_Data

## Data Availability

All simulation data is generated with the code available and described in the Zenodo repository: https://doi.org/10.5281/zenodo.12707357.

## References

[1] Creutzig F , *et al*. 2022. Demand, services and social aspects of mitigation. In: Shukla PR, Skea J, Slade R, Al Khourdajie A, van Diemen R, McCollum D, Pathak M, Some S, Vyas P, Fradera R, Belkacemi M, Hasija A, Lisboa G, Luz S, Malley J, editors. Climate change 2022: mitigation of climate change. Contribution of working group III to the sixth assessment report of the intergovernmental panel on climate change, book section 5. Cambridge (UK): Cambridge University Press.

[2] Dietz T , Gardner GT, Gilligan J, Stern PC, Vandenbergh MP. 2009. Household actions can provide a behavioral wedge to rapidly reduce us carbon emissions. Proc Natl Acad Sci USA. 106(44):18452–18456. 19858494 10.1073/pnas.0908738106 PMC2767367

[3] Bak-Coleman JB , *et al*. 2021. Stewardship of global collective behavior. Proc Natl Acad Sci USA. 118(27):e2025764118. 34155097 10.1073/pnas.2025764118 PMC8271675

[4] Efferson C , Vogt S, Fehr E. 2020. The promise and the peril of using social influence to reverse harmful traditions. Nat Hum Behav. 4(1):55–68. 31792402 10.1038/s41562-019-0768-2

[5] Nyborg K , *et al*. 2016. Social norms as solutions. Science. 354(6308):42–43. 27846488 10.1126/science.aaf8317

[6] Smith SR . 2023. Understanding and acting on positive tipping points. In: The global tipping points report 2023. Exeter (UK): University of Exeter. p. 10.

[7] Gelfand MJ , Gavrilets S, Nunn N. 2024. Norm dynamics: interdisciplinary perspectives on social norm emergence, persistence, and change. Annu Rev Psychol. 75:341–378. 37906949 10.1146/annurev-psych-033020-013319

[8] Cooper R . 1999. Coordination games. Cambridge (UK): Cambridge University Press.

[9] Asch SE . 1956. Studies of independence and conformity: I. A minority of one against a unanimous majority. Psychol Monogr: General Appl. 70(9):1.

[10] Efferson C , Lalive R, Richerson PJ, McElreath R, Lubell M. 2008. Conformists and mavericks: the empirics of frequency-dependent cultural transmission. Evol Hum Behav. 29(1):56–64.

[11] Van Leeuwen EJC , Haun DBM. 2014. Conformity without majority? the case for demarcating social from majority influences. Anim Behav. 96:187–194.

[12] Boyd R , Richerson PJ. 1988. Culture and the evolutionary process. Chicago: University of Chicago Press.

[13] Morgan TJH , Laland KN. 2012. The biological bases of conformity. Front Neurosci. 6:87. 22712006 10.3389/fnins.2012.00087 PMC3375089

[14] Taylor DG . 1982. Pluralistic ignorance and the spiral of silence: a formal analysis. Public Opin Q. 46(3):311–335.

[15] Janis IL . 1972. Victims of groupthink: a psychological study of foreign-policy decisions and fiascoes.

[16] Solomon M . 2006. Groupthink versus the wisdom of crowds: the social epistemology of deliberation and dissent. South J Philos. 44(S1):28–42.

[17] Noelle-Neumann E . 1974. The spiral of silence a theory of public opinion. J Commun. 24(2):43–51.

[18] Przeworski A . 2003. Freedom to choose and democracy. Econ Philos. 19(2):265–279.

[19] Courson BD , Fitouchi L, Bouchaud J-P, Benzaquen M. 2021. Cultural diversity and wisdom of crowds are mutually beneficial and evolutionarily stable. Sci Rep. 11(1):16566. 34400679 10.1038/s41598-021-95914-7 PMC8368188

[20] Santos FP , Levin SA, Vasconcelos VV. 2021. Biased perceptions explain collective action deadlocks and suggest new mechanisms to prompt cooperation. Iscience. 24(4):102375. 33948558 10.1016/j.isci.2021.102375 PMC8080528

[21] Vriens E , Tummolini L, Andrighetto G. 2023. Vaccine-hesitant people misperceive the social norm of vaccination. PNAS Nexus. 2(5):pgad132. 37168670 10.1093/pnasnexus/pgad132 PMC10165803

[22] Ettinger J , *et al*. 2023. Breaking the climate spiral of silence: lessons from a COP26 climate conversations campaign. Clim Change. 176(3):22.

[23] Geiger N , Swim JK. 2016. Climate of silence: pluralistic ignorance as a barrier to climate change discussion. J Environ Psychol. 47:79–90.

[24] Bursztyn L , Egorov G, Fiorin S. 2020. From extreme to mainstream: the erosion of social norms. Am Econ Rev. 110(11):3522–3548.

[25] Brinkman RL , Brinkman JE. 1997. Cultural lag: conception and theory. Int J Soc Econ. 24(6):609–627.

[26] Priest SH , Ten Eyck T. 2004. Transborder information, local resistance, and the spiral of silence: biotechnology and public opinion in the United States. In: Biotechnology and communication. Oxfordshire (UK): Routledge. p. 191.

[27] Sunstein CR . 2002. Conformity and dissent.

[28] Denton KK , Liberman U, Feldman MW. 2021. On randomly changing conformity bias in cultural transmission. Proc Natl Acad Sci USA. 118(34):e2107204118. 34417299 10.1073/pnas.2107204118 PMC8403876

[29] Hornsey MJ , Majkut L, Terry DJ, McKimmie BM. 2003. On being loud and proud: non-conformity and counter-conformity to group norms. Br J Soc Psychol. 42(3):319–335. 14567840 10.1348/014466603322438189

[30] Mesoudi A , Lycett SJ. 2009. Random copying, frequency-dependent copying and culture change. Evol Hum Behav. 30(1):41–48.

[31] Shennan SJ , Wilkinson JR. 2001. Ceramic style change and neutral evolution: a case study from neolithic europe. Am Antiq. 66(4):577–593.

[32] Reader SM , Laland KN. 2001. Primate innovation: sex, age and social rank differences. Int J Primatol. 22:787–805.

[33] Leonardelli GJ , Pickett CL, Brewer MB. 2010. Optimal distinctiveness theory: A framework for social identity, social cognition, and intergroup relations. In: Advances in experimental social psychology, volume 43. Amsterdam (NL): Elsevier. p.63.

[34] Bramoullé Y . 2007. Anti-coordination and social interactions. Games Econ Behav. 58(1):30–49.

[35] Rapoport A , Chammah AM. 1966. The game of chicken. Am Behav Sci. 10(3):10–28.

[36] Granovetter M , Soong R. 1986. Threshold models of interpersonal effects in consumer demand. J Econ Behav Organ. 7(1):83–99.

[37] Gardikiotis A . 2011. Minority influence. Soc Personal Psychol Compass. 5(9):679–693.

[38] Jarman M , *et al*. 2015. The critical few: anticonformists at the crossroads of minority opinion survival and collapse. J Artif Soc Soc Simul. 18(1):6.

[39] Yang VC , Galesic M, McGuinness H, Harutyunyan A. 2021. Dynamical system model predicts when social learners impair collective performance. Proc Natl Acad Sci USA. 118(35):e2106292118. 34446556 10.1073/pnas.2106292118 PMC8536379

[40] Couzin ID , *et al*. 2011. Uninformed individuals promote democratic consensus in animal groups. Science. 334(6062):1578–1580. 22174256 10.1126/science.1210280

[41] Gavrilets S . 2020. The dynamics of injunctive social norms. Evol Hum Sci. 2:e60. 37588350 10.1017/ehs.2020.58 PMC10427483

[42] Yang L , *et al*. 2022. Sociocultural determinants of global mask-wearing behavior. Proc Natl Acad Sci USA. 119(41):e2213525119. 36191222 10.1073/pnas.2213525119 PMC9565043

[43] Scheffer M , *et al*. 2012. Anticipating critical transitions. Science. 338(6105):344–348. 23087241 10.1126/science.1225244

[44] Centola D . 2018. How behavior spreads: the science of complex contagions. Princeton: Princeton University Press Vol. 3.

[45] Granovetter M . 1978. Threshold models of collective behavior. Am J Sociol. 83(6):1420–1443.

[46] Juul JS , Porter MA. 2019. Hipsters on networks: how a minority group of individuals can lead to an antiestablishment majority. Phys Rev E. 99(2):022313. 30934370 10.1103/PhysRevE.99.022313 PMC7217548

[47] Claidière N , Whiten A. 2012. Integrating the study of conformity and culture in humans and nonhuman animals. Psychol Bull. 138(1):126. 22061691 10.1037/a0025868

[48] Centola D , Becker J, Brackbill D, Baronchelli A. 2018. Experimental evidence for tipping points in social convention. Science. 360(6393):1116–1119. 29880688 10.1126/science.aas8827

[49] Galam S . 2004. Contrarian deterministic effects on opinion dynamics: “the hung elections scenario”. Physica A. 333:453–460.

[50] Grabisch M , Li F. 2020. Anti-conformism in the threshold model of collective behavior. Dyn Games Appl. 10(2):444–477.

[51] Nowak B , Grabisch M, Sznajd-Weron K. 2022. Threshold model with anticonformity under random sequential updating. Phys Rev E. 105(5):054314. 35706317 10.1103/PhysRevE.105.054314

[52] Nowak B , Sznajd-Weron K. 2019. Homogeneous symmetrical threshold model with nonconformity: independence versus anticonformity. Complexity. 2019:5150825.

[53] Prasetyo J , De Masi G, Ferrante E. 2019. Collective decision making in dynamic environments. Swarm Intell. 13(3–4):217–243.

[54] Andreoni J , Nikiforakis N, Siegenthaler S. 2017. Social change and the conformity trap. *Unpublished manuscript*. https://www.aeaweb.org/conference/2018/preliminary/paper/rdds26N9.

[55] Everall JP , Donges JF, Otto IM. 2023. The pareto effect in tipping social networks: from minority to majority. EGUsphere. 2023:1–38.

[56] Andreoni J , Nikiforakis N, Siegenthaler S. 2021. Predicting social tipping and norm change in controlled experiments. Proc Natl Acad Sci USA. 118(16):e2014893118. 33859043 10.1073/pnas.2014893118 PMC8072257

[57] Chenoweth E , Belgioioso M. 2019. The physics of dissent and the effects of movement momentum. Nat Hum Behav. 3(10):1088–1095. 31384022 10.1038/s41562-019-0665-8

[58] Gavrilets S , Richerson PJ. 2017. Collective action and the evolution of social norm internalization. Proc Natl Acad Sci USA. 114(23):6068–6073. 28533363 10.1073/pnas.1703857114 PMC5468620

[59] Zhang K , Yang Z, Başar T. 2021. Multi-agent reinforcement learning: a selective overview of theories and algorithms. In: Handbook of reinforcement learning and control. Cham: Springer. p. 321–384.

[60] Brouwer C , Bolderdijk J-W, Cornelissen G, Kurz T. 2022. Communication strategies for moral rebels: how to talk about change in order to inspire self-efficacy in others. Wiley Interdiscip Rev: Clim Change. 13(5):e781.

[61] Ehret S , Constantino SM, Weber EU, Efferson C, Vogt S. 2022. Group identities can undermine social tipping after intervention. Nat Hum Behav. 6(12):1669–1679. 36138223 10.1038/s41562-022-01440-5

[62] Li A , *et al*. 2020. Evolution of cooperation on temporal networks. Nat Commun. 11(1):2259. 32385279 10.1038/s41467-020-16088-w PMC7210286

[63] Tokita CK , Guess AM, Tarnita CE. 2021. Polarized information ecosystems can reorganize social networks via information cascades. Proc Natl Acad Sci USA. 118(50):e2102147118. 34876511 10.1073/pnas.2102147118 PMC8685718

[64] Vasconcelos VV , *et al*. 2021. Segregation and clustering of preferences erode socially beneficial coordination. Proc Natl Acad Sci USA. 118(50):e2102153118. 34876514 10.1073/pnas.2102153118 PMC8685719

[65] d’I Treen KM , Williams HTP, O’Neill SJ. 2020. Online misinformation about climate change. Wiley Interdiscip Rev: Clim Change. 11(5):e665.

[66] Kelly D , Westra E. Why does moral progress feel preachy and annoying?—Aeon Essays—aeon.co; 2024 [Accessed 2024 Jun 28]. https://aeon.co/essays/why-does-moral-progress-feel-preachy-and-annoying.

[67] Gelfand MJ , *et al*. 2011. Differences between tight and loose cultures: a 33-nation study. Science. 332(6033):1100–1104. 21617077 10.1126/science.1197754

[68] Mittal D , Vasconcelos VV, Constantino SM. Model implementation of: anti-conformists catalyze societal transitions and facilitate the expression of evolving preferences. July 2024, doi: 10.5281/zenodo.12707357. 10.1093/pnasnexus/pgae302 PMC11302527 39108299

[69] Szabó G , Tőke C. 1998. Evolutionary prisoner’s dilemma game on a square lattice. Phys Rev E. 58(1):69.

[70] Xulvi-Brunet R , Sokolov IM. 2004. Reshuffling scale-free networks: from random to assortative. Phys Rev E. 70(6):066102. 10.1103/PhysRevE.70.066102 15697429

