# Supplementary material for: Anticonformists catalyze societal transitions and facilitate the expression of evolving preferences
Source: PNAS Nexus. 2024 Jul 25;3(8):pgae302. doi: 10.1093/pnasnexus/pgae302 (PMC11302527; doi:10.1093/pnasnexus/pgae302)
Supplement: pgae302_Supplementary_Data [file pgae302_supplementary_data.pdf]

PNAS nexus

Supporting Information for

Anti-conformists catalyze societal transitions and facilitate the expression of evolving preferences

Dhruv Mittal*, Sara M. Constantino and Vítor V. Vasconcelos*

***Correspondence to: Dhruv Mittal, d.mittal@uva.nl, and Vítor V. Vasconcelos, v.v.vasconcelos@uva.nl**

This PDF file includes:

Supporting text
Figs. S1 to S18
SI References

Contents

1 Game-theoretic framework of collective decision-making **3**
A Linear frequency dependency in social influence . . . 3
B Higher order frequency dependency in social influence . . . 4

2 Non-conformity **5**

3 Markov chain analysis **6**

4 Effects due to spatial correlations and finite size **7**

5 Measures of social well-being **9**
A Alignment choice and preference . . . 9
B Satisfaction . . . 9
C Social pressure . . . 9
D Time taken for social tipping in case of linearly changing preferences . . . 10
E Social Welfare . . . 10

6 Additional supplementary figures (Figures S14-S18) **12**

Supporting Information Text

1. Game-theoretic framework of collective decision-making

We present a framework that contextualizes the threshold model understanding of collective behavior in a game theoretic setting (1). We assume that an individual's decision-making relies on two aspects: their personal evaluation of the choices they face and the socio-psychological and economic considerations deriving from their neighbors' choices. The dependence of the utility on the social environment can be broken down into the dependence on the frequency of that choice, controlled by ${w_i}^c$, and the frequency of the alternate choices in the immediate neighborhood, controlled by ${w_i}^{\bar{c}}$.

$$ {U_i}^A = {o_i}^A + {w_i}^c F({x_i}^A, k_i) + {w_i}^{\bar{c}} G({x_i}^B, k_i) \quad \text{[S1]} $$

$$ {U_i}^B = {o_i}^B + {w_i}^c F({x_i}^B, k_i) + {w_i}^{\bar{c}} G({x_i}^A, k_i) \quad \text{[S2]} $$

Here, ${x_i}^A({x_i}^B)$ is the fraction of neighbors with choice A(B), and k_i is the number of neighbors. When the net social influence to choose option 'A' is positively dependent on the relative frequency of choice 'A', the behavior can be considered to be conforming, and, conversely, anti-conforming when this dependence is negative. This framework can be extended to consider more than two choices.

A. Linear frequency dependency in social influence. For the purposes of this study, which focuses on the expression of preferences, in the main text, we use a model that assumes a linear frequency dependency where $F(x, k) = G(x, k) = kx$. The conformists (anti-conformists) are assigned a higher (lower) weight to align with the choice than aligning with the alternative, $w^c = 1.5, w^{\bar{c}} = 0.5$ ($w^c = 0.5, w^{\bar{c}} = 1.5$). We define $(w^c - w^{\bar{c}})$ as conformity w, with conformists having $w = 1$ and anti-conformists having $w = -1$. With agents preferring either A or B, we end up with four different types of response functions in the population (Fig. S1). Conforming agents have a threshold of how many A's they must observe in their neighborhood before they switch to A, while anti-conformists have a threshold beyond which they abandon choice A. Their net preference moves that threshold. These two kinds of thresholds are similar to Grannovetter and Soong's lower and upper thresholds (2). In our model, the conformists can be thought of as those having only a lower threshold, while anti-conformists can be thought of as instigators, that is, those who only have an upper threshold.

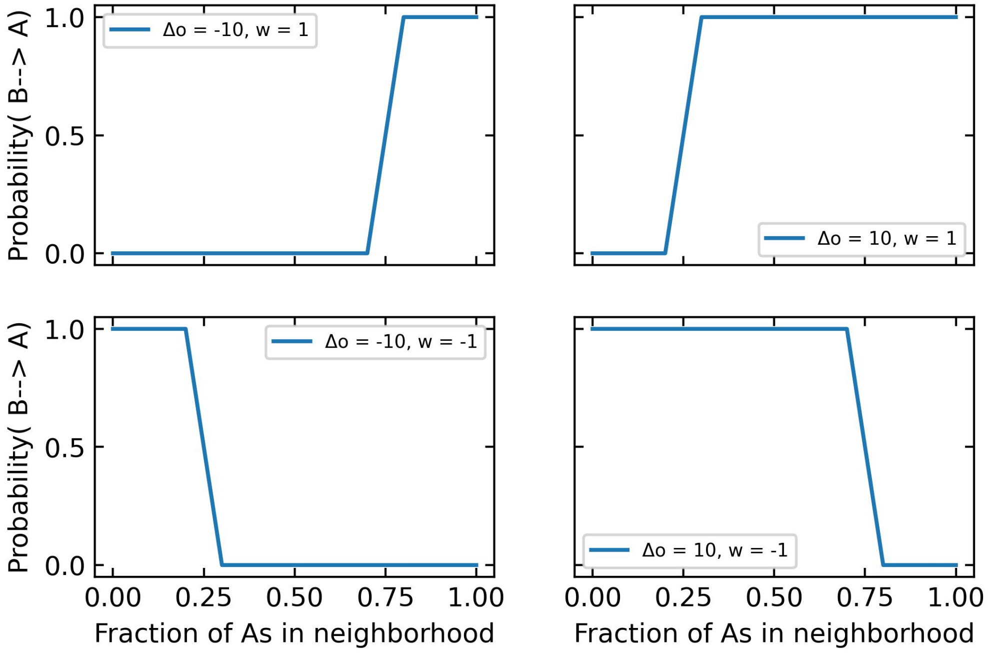

Fig. S1. The probability of switching from choice B to A for agents with a net preference for A ($\Delta o = 10$) or B ($\Delta o = -10$) and degree $k = 20$, who can be either conforming, ($w^c = 1.5$, $w^{\bar{c}} = 0.5$, i.e., $w = 1$) or anti-conforming ($w^c = 0.5$, $w^{\bar{c}} = 1.5$, i.e., $w = -1$). The response functions do not depend on whether the neighbors are conformists or anti-conformists or their preferences since those are not considered visible.

B. Higher order frequency dependency in social influence. Considering non-linear dependencies can also make for nuanced behavior that combines forward and reverse bandwagon effects like demand and supply that manifest in complex behavioral responses as a function of the local neighborhood (2). Effectively, this leads to individuals having more than one threshold to adopt or reject an option. In the following section, we model another type of agent, which we call a conditional anti-conformist. Such agents anti-conform only when a choice starts completely dominating in their social environment while conforming in more polymorphic setups.

$$F(x,k) = G(x,k) = k^3(x-a)(x-b)(x-c) \quad \text{[S3]}$$

For suitable values of w^c and $w^{\bar{c}}$ such that $w^c - w^{\bar{c}} < 0$, we get response functions as shown in Fig.S2.

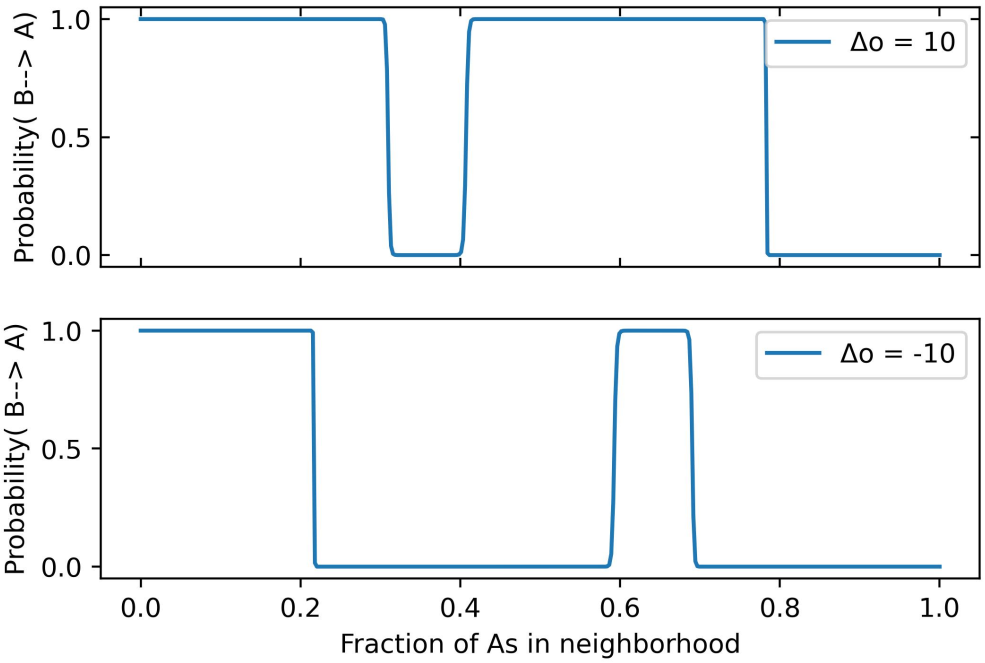

Fig. S2. The probability of switching from choice B to A for conditional anti-conformists with a net preference for A ($\Delta o = 10$) or B ($\Delta o = -10$).

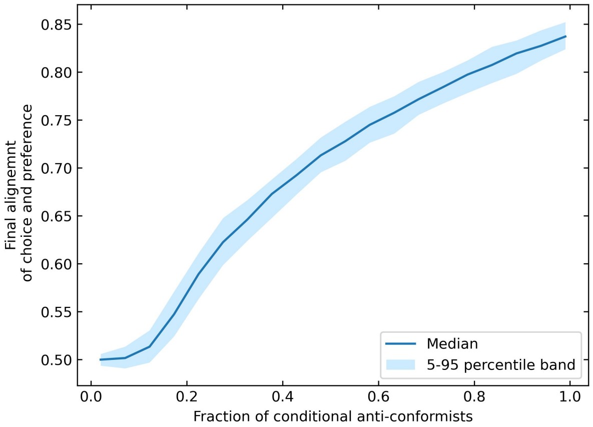

Fig. S3. Expression of preferences with conditional anti-conformists: The average equilibrium alignment of choice and preference is plotted for a heterogeneous population comprising half of the individuals preferring A and the other half preferring B. Agents were placed on a Barabási-Albert network with $k_{min} = 20$. A). We see a weak non-linear continuous response to the fraction of conditional anti-conformists in the population.

This behavior can be thought of as weaker anti-conformity, in the sense that individuals will still change behavior when they adopt a strictly dominant local behavior (i.e., the probability of changing from B to A is 1 when everyone is a B and is 0 when everyone is an A). This type of behavior also facilitates the expression of preferences and enhances adaptability in changing environments. However, weaker dissent in the face of the status quo means that more such individuals are required to make significant differences in population-level behavior as seen in figure S3, and also in dynamic environments as in figures S4 and S5.

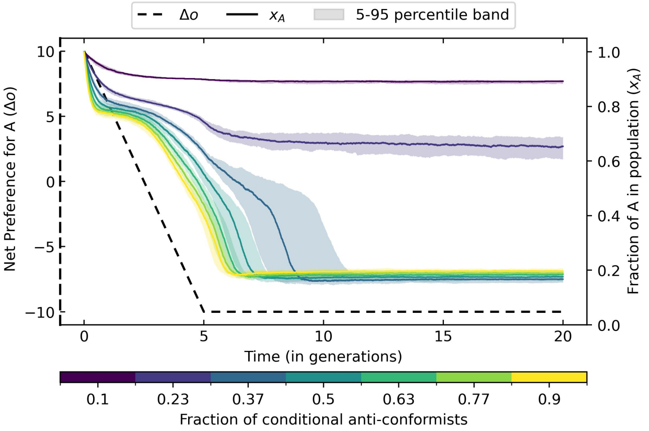

Fig. S4. Population with conditional anti-conformists in dynamic environments: The net preference for choice A (Δo) decreases linearly with time. We plot the median trajectory of the fraction of choice A in the population on Barabási-Albert networks over time. We vary the fraction of anti-conformists in the population. We see a faster societal transition from choice A to B as the fraction of conditional anti-conformists increases. The population size is fixed at 1000.

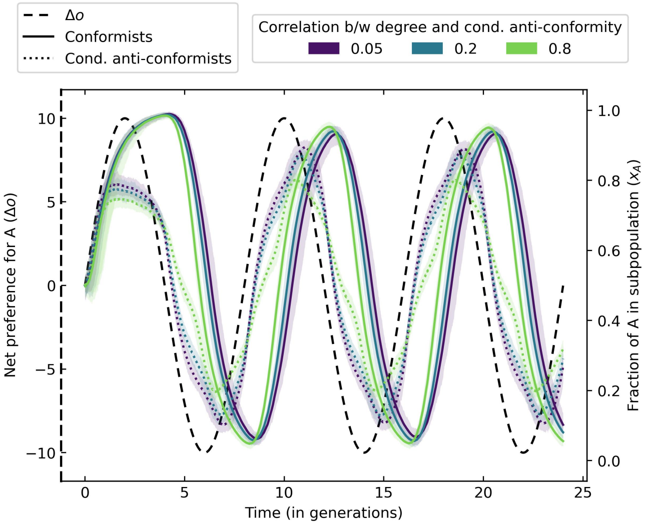

Fig. S5. Population with conditional anti-conformists in a fluctuating environments: The net preference for choice A (Δo) fluctuates sinusoidally with time. We plot the median trajectory of the fraction of different sub-populations, i.e., the conformists (solid lines) and conditional anti-conformists (dotted lines), picking choice A over time. Colors indicate populations that vary in the value of the correlation between degree and anti-conformity, keeping the fraction of conditional anti-conformists equal to 0.2. The population size considered is fixed at $N = 1000$.

2. Non-conformity

In this section, we look at the effect of non-conformists on collective decision-making. Such individuals remain completely unaffected by social pressures, and they only factor their personal preferences into the decision-making utility (equivalent to setting w^c and $w^{\bar{c}}$ to zero). These agents can be thought of as stubborn, committed, or moral rebels (3, 4).

When it comes to the expression of preferences, a population requires twice as many non-conformists as compared to anti-conformists (Fig.S6). Further, even with central placement, a population needs around 15 percent of non-conformists as compared to 5 percent of anti-conformists in a population, for the same setup. This puts into perspective the effectiveness of anti-conformists.

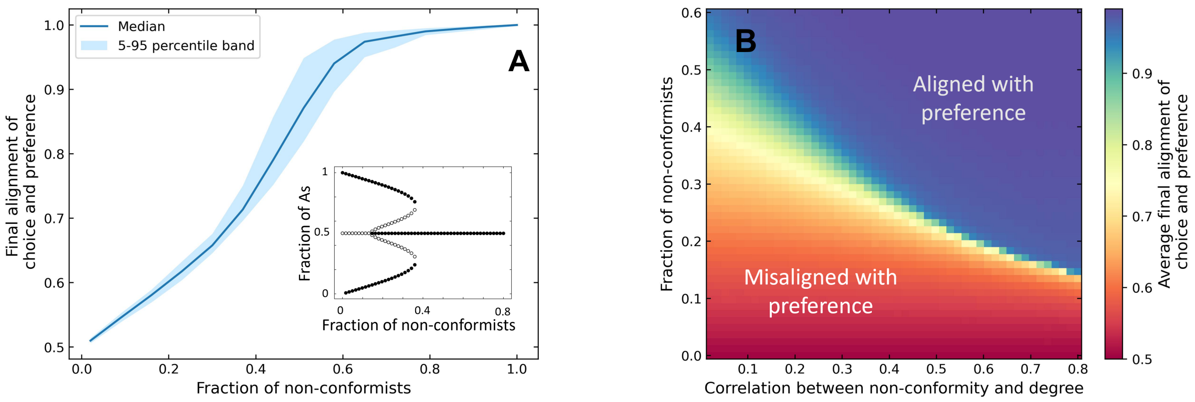

Fig. S6. Effect of non-conformity on expression of preferences: The average equilibrium alignment of choice and preference is plotted for a heterogeneous population comprising half of the individuals preferring A and the other half preferring B. Agents were placed on a Barabási-Albert network with $k_{min} = 20$. A) For the heterogeneous BA network, there is a weak non-linear continuous response to the fraction of anti-conformists in the population driven by local network effects, for a low centrality. In networks without spatial correlations with probabilistic connections (inset), the transition is always discontinuous in the fraction of non-conformists. B) A smooth phase transition is seen from a state of misalignment between the choices and preferences to a state of alignment in the parameter space as centrality and fraction of anti-conformists increase when their centrality is low, but it becomes more abrupt when that centrality increases. Populations with a low fraction of non-conformists need them to be more central in the network to achieve alignment between choice and preference. The simulations are done for a population size of 1000 with 50 different network realizations

Further, in a fluctuating environment, non-conformists do not emerge as effective leaders of change, as the conformists in the population lag with respect to the changing environment, as seen in Fig.S7.

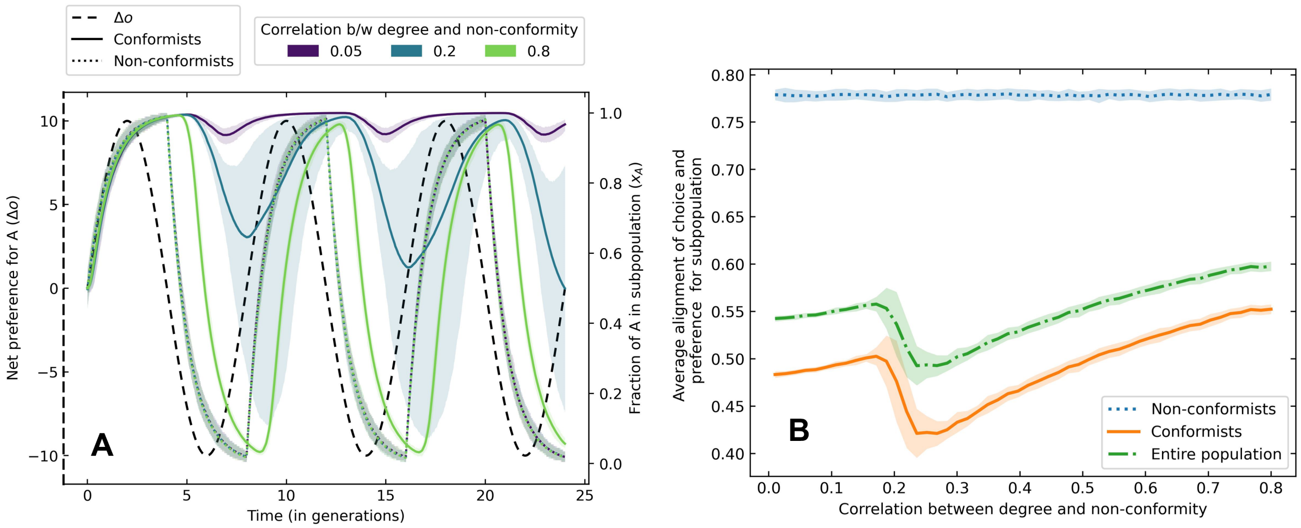

Fig. S7. Non-conformists in fluctuating environments: The net preference for choice A (Δo) fluctuates sinusoidally with time. In the top panel, we plot the median trajectory of the fraction of different sub-populations, i.e., the conformists (solid lines) and non-conformists (dotted lines), picking choice A over time. Colors indicate populations that vary in the value of the correlation between degree and non-conformity, keeping the fraction of non-conformists equal to 0.2. The non-conformists switch their choices to alternative behavior or technology only when it is profitable. The bottom panel shows the average alignment between the choices and preferences of the two sub-populations and for the entire population over time as a function of differing degree centrality of non-conformists in the population. We see that the alignment of choice and preference for conformists in the presence of centrally placed non-conformists is much lower compared to populations with centrally placed anti-conformists (Fig. 4). The population size considered is fixed at $N = 1000$.

3. Markov chain analysis

We employ the Markov chain analysis to understand the population dynamics of collective decision-making in the well-mixed limit. Let x be the fraction of individuals choosing A. Since at each timestep, we consider a single update, the transition probability, T^{A+}, represents the probability that the system goes from x to $x + 1/N$ in one update and is the chance of an individual currently choosing B chooses A in the next timestep. The probability that an agent with degree k (the number of

neighbors that the focal agent samples randomly from the entire population) chooses option A at any given time is:

$$P_A^{kwo}(x) = \sum_{j=0}^{k} \binom{k}{j} p_{kj}^{A\,wo} x^j (1-x)^{k-j}. \quad \text{[S4]}$$

where $p_{kj}^{A\,wo}$ is the probability of the agent with preference o and conformity w choosing A given that it has k neighbors out of which j have made choice A (equation for p^A is given in Materials and Methods in the main text) and x is the fraction of agents in the population making choice A. Similarly to the ABM, all agents have an equal likelihood of being selected at any given time, and we assume an independent distribution agent attributes preference (p_o), conformity(p_w) and degree (p_k) which corresponds to the case of random placement of preferences and conformity on the network. This gives us:

$$T^{A+} = (1-x) \int\int\int p_o p_w p_k P_A^{kwo}(x) do dw dk. \quad \text{[S5]}$$

Similarly,

$$T^{A-} = x \int\int\int p_o p_w p_k (1 - P_A^{kwo}(x)) do dw dk. \quad \text{[S6]}$$

In the limit of large populations, the process X becomes continuous and can be approximated by a stochastic differential equation (5). Further, the diffusion term vanishes, and we get the ODE for x as the balance of $T^{A+} - T^{A-}$ the probability of picking an agent who already has chosen A, which is the same as x

$$\begin{aligned}
\dot{x} &= T^{A+} - T^{A-} \quad \text{[S7]}\\
&= (1-x) \int\int\int p_o p_w p_k P_A^{kwo}(x) do dw dk - x \int\int\int p_o p_w p_k (1 - P_A^{kwo}(x)) do dw dk \\
&= \int\int\int p_o p_w p_k P_A do dw dk - x \int\int\int p_o p_w p_k P_A do dw dk - x \int\int\int p_o p_w p_k do dw dk + x \int\int\int p_o p_w p_k (P_A) do dw dk \\
&= \int\int\int p_o p_w p_k P_A^{kwo}(x) do dw dk - x.
\end{aligned}$$

From this equation, we can find the fixed points for the system (panel A in Fig. S8 and S10)

4. Effects due to spatial correlations and finite size

The dynamics of collective behavior on a graph can be affected by spatial correlations, locality of interactions, and finite systems. These factors are not accounted for in the Markov chain analysis, which is applied in the well-mixed large system size. To check for the impact of spatial correlations and locality of interactions, we compare the results of dynamics on graphs to the results of a system without the locality of interactions but maintaining the degree distributions similar to that of the graphs. In this setup, the agents randomly sample agents from the entire population whenever they make a decision, the sample size being equivalent to the degree on a graph. This mimics a well-mixed limit.

We see that for a highly heterogeneous network, i.e., the Barabási-Albert network, the effect of the locality of interactions is very pronounced (Fig. S8). We also see that the effect of system size on equilibrium is quite limited (Fig. S9). The effect of locality is not as pronounced in a more homogeneous network, i.e., the Erdős-Rényi network (Fig. S10).

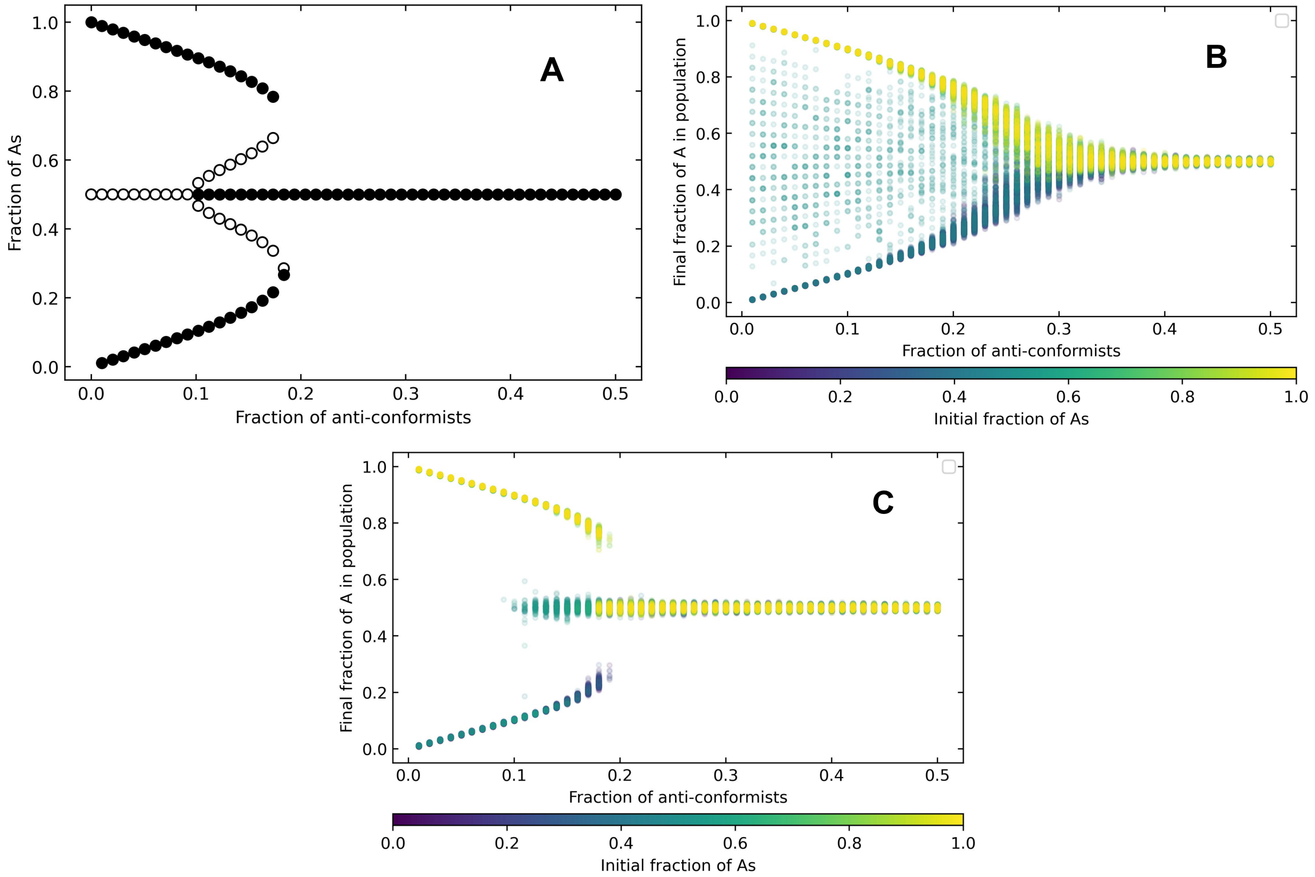

Fig. S8. Equilibrium for heterogeneous networks: A) The stable fixed points (solid circles) and unstable fixed points (empty circles) are calculated using Markov chain analysis. The equilibrium fraction of A's in the population is plotted for populations connected by a Barabási-Albert network in plot B and connected probabilistically with a power law degree distribution in plot C. Plot C is in better agreement with the bifurcation analysis in A than plot B. This highlights the effect of locality of interactions. The population size is fixed at 2000.

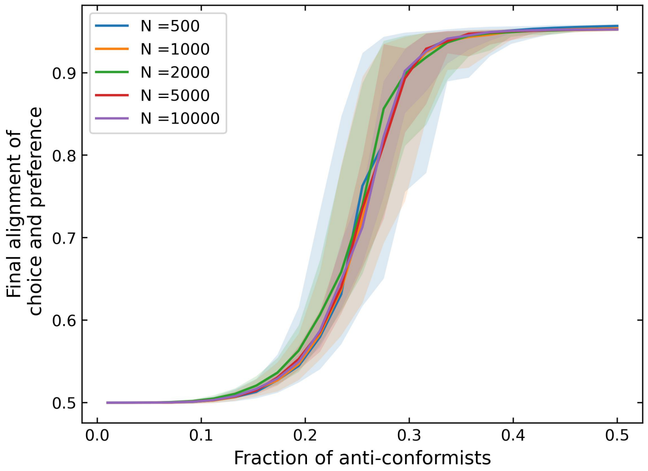

Fig. S9. Finite size effects: The equilibrium alignment between choice and preference for the population is plotted against the fraction of anti-conformists for different population sizes (N). We see minimal effects of finite size in determining the critical fraction of anti-conformists. The 5-95 percentile band is represented by the shaded region for each population size. This spread decreases as the population size increases.

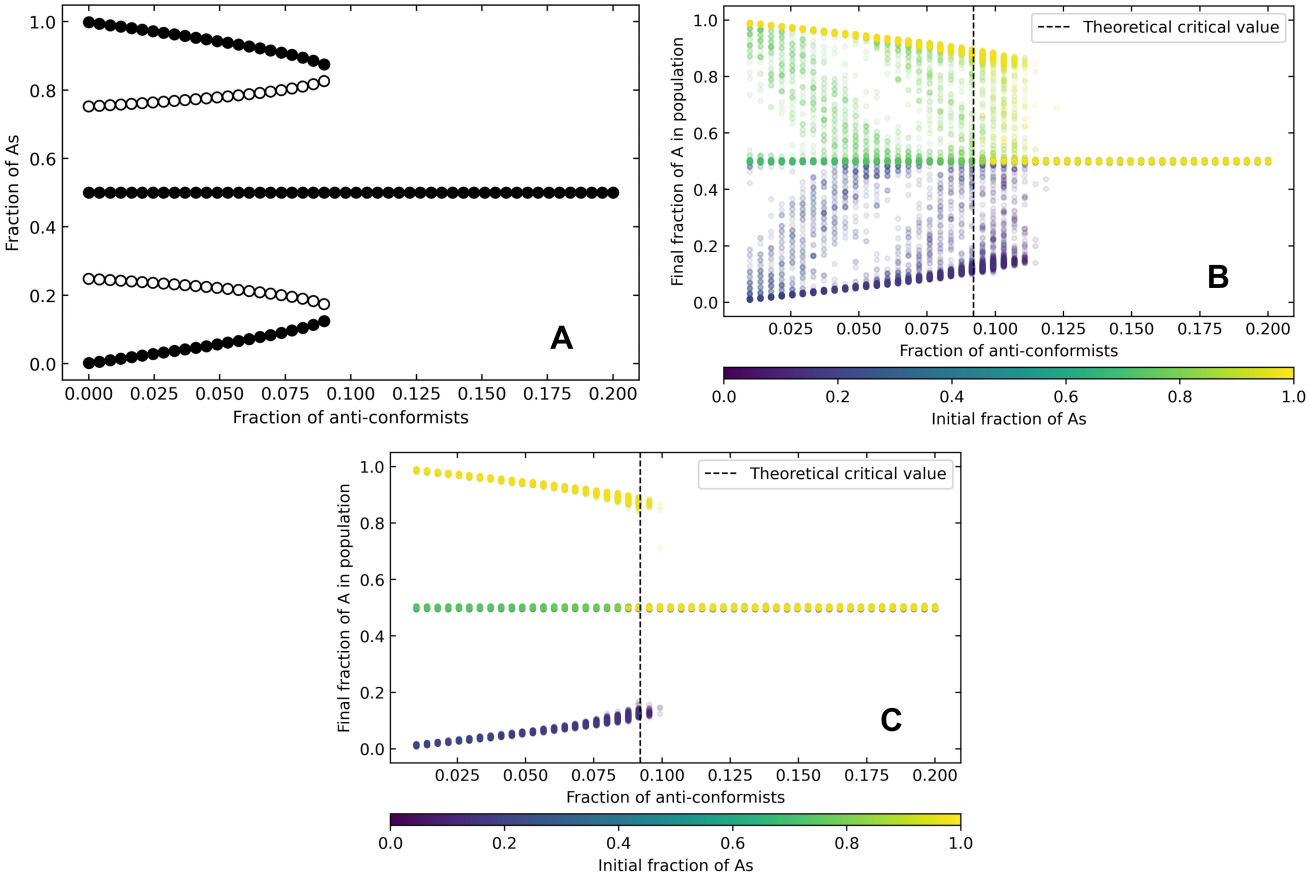

Fig. S10. Equilibrium for homogeneous networks: The analysis is done for a heterogeneous population comprising half of the individuals preferring A ($\Delta o = 15$) and the other half preferring B ($\Delta o = -15$) with average degree $\langle k \rangle = 30$. A) The stable fixed points (solid circles) and unstable fixed points (empty circles) are calculated using Markov chain analysis. The equilibrium fraction of A's in the population is plotted for populations connected by an Erdős-Rényi network in plot B and connected probabilistically with a normal degree distribution in plot C. Plot C is in better agreement with the bifurcation analysis in A than plot B. This highlights the effect of locality of interactions. Compared to a heterogeneous network (Fig S8b), dynamics on a homogeneous network can be approximated much better by Markov chain analysis. The population size is fixed at 2000.

5. Measures of social well-being

In this section, we talk about the measures we have used to quantify the notion of social well-being in the framework of collective decision-making we have presented.

A. Alignment choice and preference. If an agent's choice, i.e., A or B (represented by 1 and -1 respectively), has the same sign as their net preference Δo_i (which is positive (negative) for A(B)), we define that as alignment between choice and preference. By counting all such agents, we get the alignment of choices and preferences in the population at any given time.

B. Satisfaction. We define the satisfaction of an agent as the marginal utility of its updated choice at the time of decision-making. So, for an agent i with choice 'A' ('B'), the satisfaction is given by ΔU_i $(-\Delta U_i)$. A list of individual satisfaction for all the agents is stored, and the mean of this list gives the average satisfaction that the population has with its current choices at any given time.

$$satisfaction = \sum_{i=1}^{N} s_i(\Delta o_i + w_i \sum_{j=1}^{N} a_{ij} s_j) \tag{S9}$$

C. Social pressure. The social pressure to choose option 'A' experienced individually by all N agents is stored in a list. The social pressure on agent i, which is equal to $w_i(\#^A - \#^B)$, The mean of this list at any point indicates the average social pressure experienced by the population at that time to choose 'A'.

$$social\ pressure = \sum_{i=1}^{N} w_i \sum_{j=1}^{N} a_{ij} s_j \tag{S8}$$

D. Time taken for social tipping in case of linearly changing preferences. The point in time of social tipping is approximated by looking for the largest variability in the fraction of a choice over a moving window in the time series.

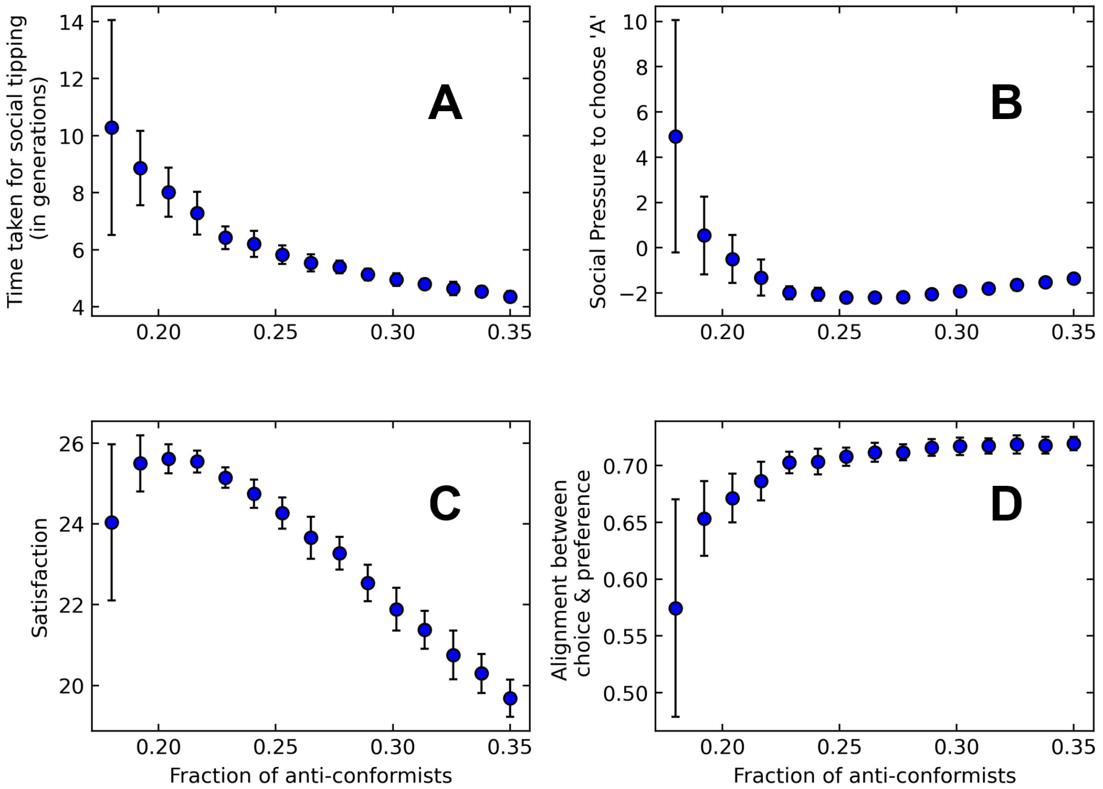

Fig. S11. Optimal level of anti-conformity in dynamic environments: We look at different variables for the population with varying fractions of anti-conformists seen in Fig 3a. A) The time taken for the population to undergo social transition decreases non-linearly as the fraction of anti-conformists increases. B) The average social pressure to choose option A over time decreases sharply with an increase in the number of anti-conformists. C) The average satisfaction of the population with its choices reaches a maximum for a fraction of anti-conformists ~ 0.20. It decreases as the fraction of anti-conformists increases beyond that. D) The average alignment of the population with the changing preferences increases and plateaus as the fraction of anti-conformists increases. We can get a sense of an optimal fraction of anti-conformists, which optimizes desired measures.

E. Social Welfare. We define welfare as the utility that an agent derives from its current choice. The population average of individual welfare gives us a measure of social welfare.

$$welfare = \sum_{i=1}^{N} (\delta_{1s_i} U^A + \delta_{-1s_i} U^B) \qquad \text{[S10]}$$

It is important to distinguish it from decision satisfaction, which considers the marginal utility of the current choice. While satisfaction pertains to the decision-making process, welfare pertains to the utility derived from the choice one has made. Welfare can be broken down into the utility of alignment of choice and inherent preference, the utility of alignment with others, and the utility of misalignment with others. This interpretation of welfare is agnostic of the agents' conformity.

Welfare is dependent on the values of w^c and $w^{\bar{c}}$. So different combinations of w^c and $w^{\bar{c}}$ such that $w^c - w^{\bar{c}} = w$ will have differing values of social welfare for identical decision-making. We plot the welfare for two such different combinations (Fig. S12). In both scenarios, an increase in the number of anti-conformists results in the utility of expressing one's preference gaining greater significance in an individual's welfare compared to the importance of social validation.

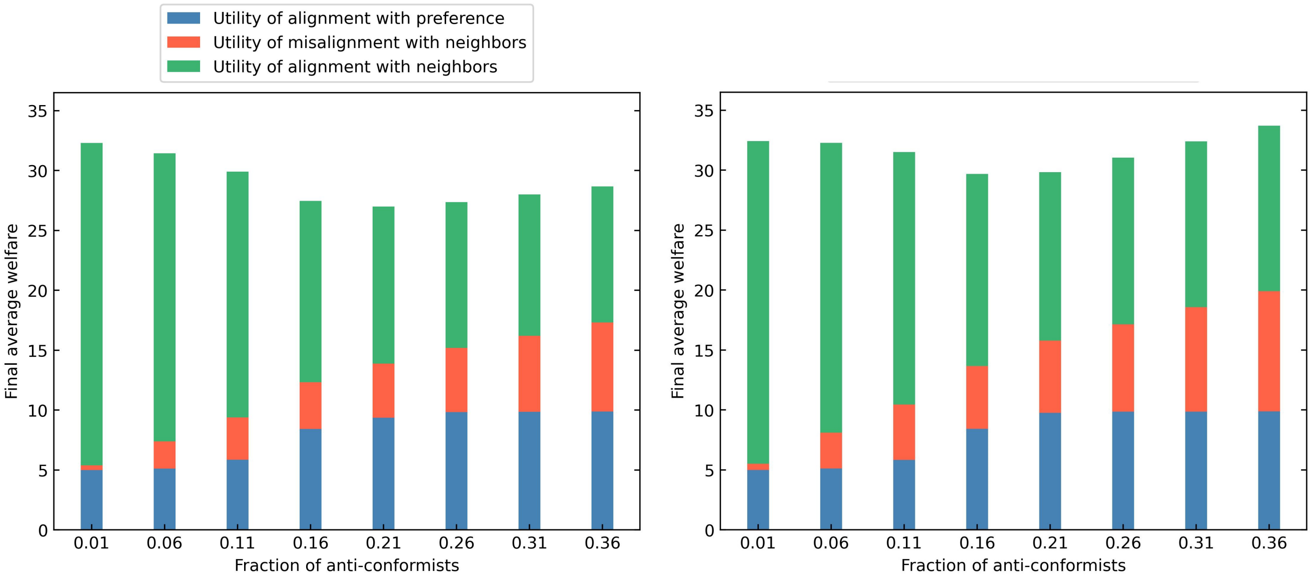

Fig. S12. Effect of anti-conformists on social welfare A) The average social welfare at equilibrium is plotted for a heterogeneous population comprising half of the individuals preferring A ($\Delta o = 10$) and the other half preferring B ($\Delta o = -10$). Agents were placed on a Barabási-Albert network with min k = 20. We vary the fraction of anti-conformists in the population. The conformists have $w^c = 0.7, w^{\bar{c}} = 0$. In the left panel, the anti-conformists have $w^c = 0.35, w^{\bar{c}} = 1.05$ and in the right panel, $w^c = 0.7, w^{\bar{c}} = 1.4$. The population size is fixed at 1000. We consider two populations with identical decision-making while showing different social welfare (welfare depends on the individual's w^c and $w^{\bar{c}}$ while the decision-making only depends on the difference between the two).

Further, in Fig. S13, we show the evolution of social welfare corresponding to the decision-making dynamics in the case of linearly changing preferences shown in Fig. 3A in the main text. The population that has a sufficient number of anti-conformists is able to achieve greater social welfare by transitioning to the optimal choice. The social welfare dips during the social transition but recovers as the population is able to coordinate on the new optimal choice. On the other hand, the social welfare for an almost completely conforming population remains stagnant as they remain stuck in the conformity trap.

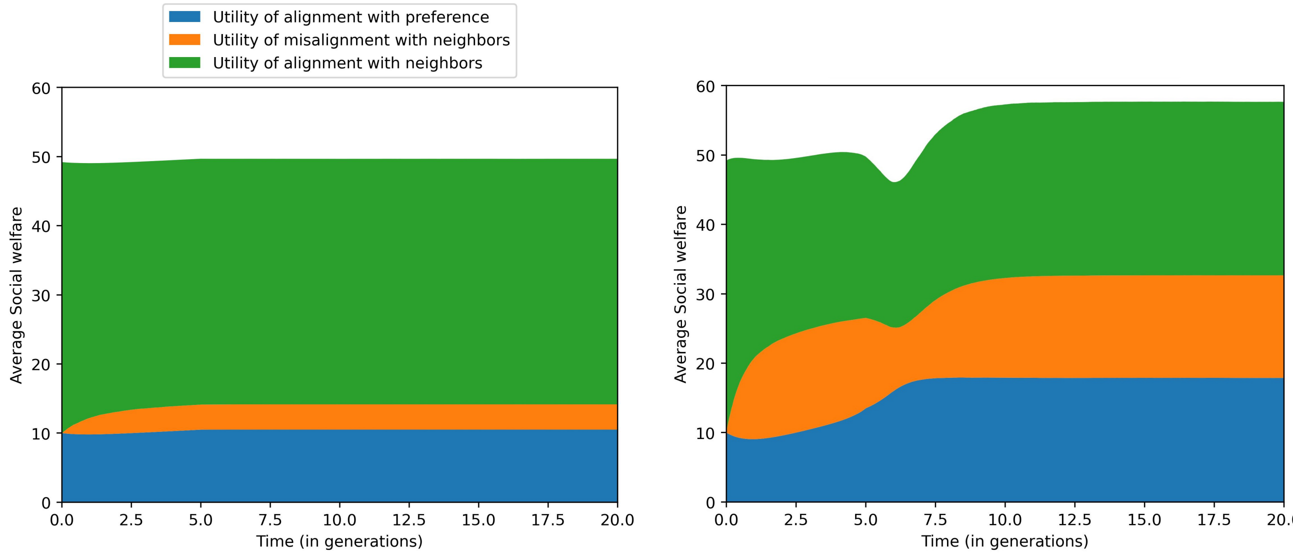

Fig. S13. Evolution of social welfare in a dynamic environment The social welfare of the population is plotted over time in the case of linearly changing preferences shown in Fig. 3A in the main text. We show the breakup of welfare into the utility arising from expressing preference, coordination, and miscoordination. o_A remains 10 while o_B increases from 0 to 20 resulting in the decrease of Δo. The conformists have $w^c = 1.0, w^{\bar{c}} = 0$, and the anti-conformists have $w^c = 1.0, w^{\bar{c}} = 2.0$. The fraction of anti-conformists is 0.05 in the left panel and 0.25 in the right panel. The population size is fixed at 1000.

6. Additional supplementary figures (Figures S14-S18)

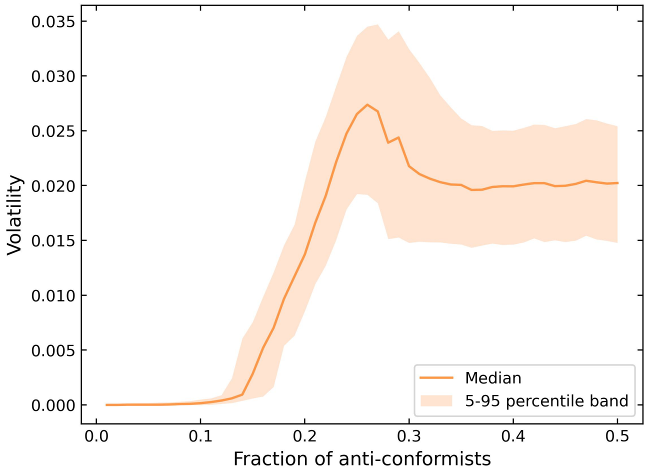

Fig. S14. Volatility due to anti-conformists in heterogeneous networks: A sharp increase in volatility is seen starting around the bifurcation points shown in Fig. S8a and remains high as the fraction of anti-conformists increases.

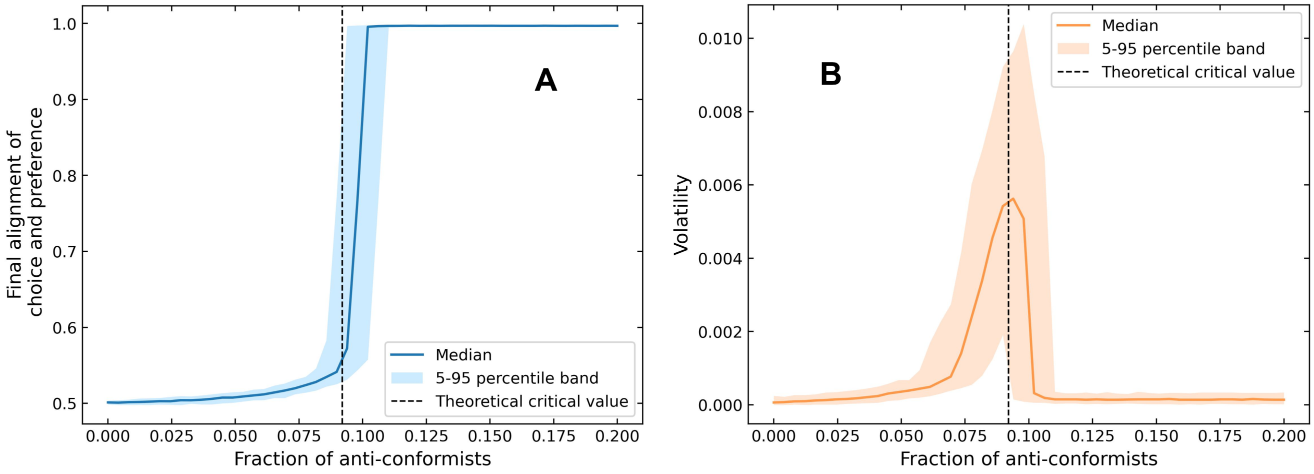

Fig. S15. Preference expression and volatility on homogeneous networks A) The average equilibrium alignment of choice and preference is plotted for a heterogeneous population comprising half of the individuals preferring A ($\Delta o = 15$) and the other half preferring B ($\Delta o = -15$) for biased initial conditions i.e., when 80 percent or more of the population starts with a particular choice. Agents were placed on an Erdős-Rényi network with $\langle k \rangle = 30$. A sharp phase transition is seen in response to the fraction of anti-conformists, which corresponds well to the bifurcation analysis in Fig S10a. B) A sharp spike in volatility is seen around the critical fraction of anti-conformists. The population size is fixed at 2000.

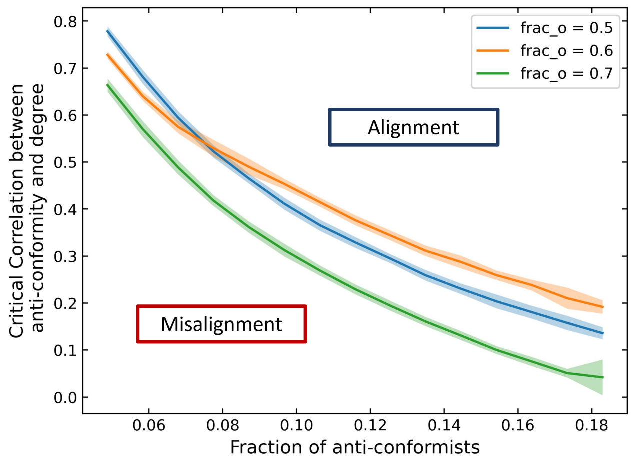

Fig. S16. Critical values of correlation between anti-conformity and degree for a given fraction of anti-conformists in the population is plotted, which separate regions of alignment and misalignment between choice and preferences. The critical values of approximated are by identifying the value of correlation corresponding to the fastest increase in alignment between choice and preference. This has been done for the fraction of the population preferring A = 0.5,0.6 and 0.7.

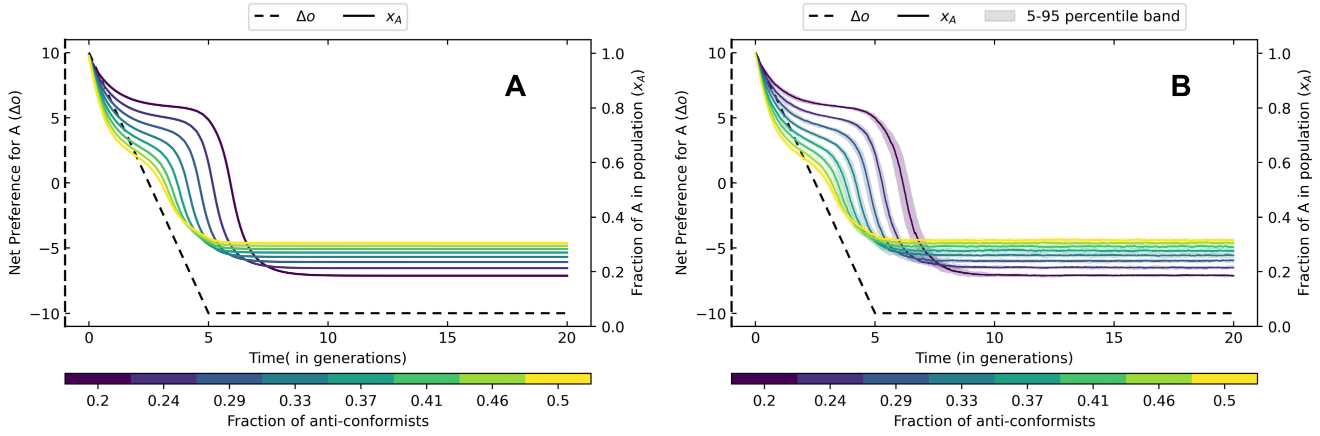

Fig. S17. Dynamic environments: The net preference for choice A (Δo) decreases linearly with time. In plot A, we plot the trajectory of the fraction of choice A (x_A) in the well-mixed large population limit for power-law degree distribution, calculated using ODE; in plot B, we plot the median trajectory of x_A in the population of size $N = 1000$ with power-law degree distribution connected probabilistically. ODE analysis gives a very good approximation of the dynamics of the probabilistically connected population but not of a population connected by a BA network due to spatial correlations.

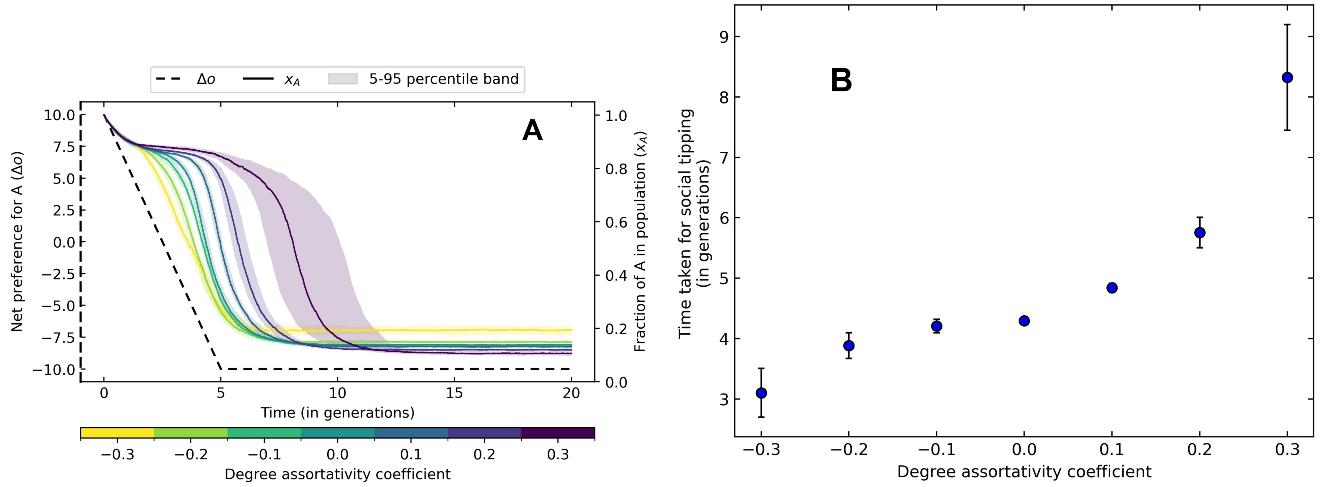

Fig. S18. Effect of degree-degree correlations in dynamic environments: The net preference for choice A (Δo) decreases linearly with time. In plot A, the median trajectory of the fraction of choice A in the population is plotted on Barabási -Albert networks with varying degree assortativity. The fraction of anti-conformists in the population is fixed at 0.15, with the correlation between degree and anti-conformity = 0.7, and the population size is fixed at 1000. In plot B, we see that populations with greater degree assortativity take longer to reach social tipping. We can conclude that centrally placed anti-conformists in a network are more effective when there is a lower degree assortativity.

References

1. Douglas Guilbeault, Joshua Becker, and Damon Centola. Complex contagions: A decade in review. *Complex spreading phenomena in social systems: Influence and contagion in real-world social networks*, pages 3–25, 2018.
2. Mark Granovetter and Roland Soong. Threshold models of interpersonal effects in consumer demand. *Journal of Economic Behavior & Organization*, 7(1):83–99, 1986.
3. Judhi Prasetyo, Giulia De Masi, and Eliseo Ferrante. Collective decision making in dynamic environments. *Swarm intelligence*, 13(3-4):217–243, 2019.
4. Claire Brouwer, Jan-Willem Bolderdijk, Gert Cornelissen, and Tim Kurz. Communication strategies for moral rebels: How to talk about change in order to inspire self-efficacy in others. *Wiley Interdisciplinary Reviews: Climate Change*, 13(5):e781, 2022.
5. Arne Traulsen, Jens Christian Claussen, and Christoph Hauert. Coevolutionary dynamics: from finite to infinite populations. *Physical review letters*, 95(23):238701, 2005.
